# Multi-omics data provide insight into the adaptation of the glasshouse plant *Rheum nobile* to the alpine subnival zone

**DOI:** 10.1038/s42003-023-05271-6

**Published:** 2023-09-04

**Authors:** Ying Li, Zhimin Niu, Mingjia Zhu, Zhenyue Wang, Renping Xu, Minjie Li, Zeyu Zheng, Zhiqiang Lu, Congcong Dong, Hongyin Hu, Yingbo Yang, Ying Wu, Dandan Wang, Jinli Yang, Jin Zhang, Dongshi Wan, Richard Abbott, Jianquan Liu, Yongzhi Yang

**Affiliations:** 1 State Key Laboratory of Grassland Agro-Ecosystems, College of Ecology, Lanzhou University, Lanzhou, 730000 China; 2 CAS Key Laboratory of Tropical Forest Ecology, Xishuangbanna Tropical Botanical Garden, Chinese Academy of Sciences, Mengla, Yunnan 666303 China; 3 School of Biology, University of St Andrews, St Andrews, Fife KY169TH UK; 4 Key Laboratory of Bio-Resource and Eco-Environment of Ministry of Education & State Key Laboratory of Hydraulics & Mountain River Engineering, College of Life Sciences, Sichuan University, Chengdu, 610065 China

**Keywords:** Evolutionary ecology, Plant evolution, Genome evolution, Molecular ecology, Abiotic

## Abstract

Subnival glasshouse plants provide a text-book example of high-altitude adaptation with reproductive organs enclosed in specialized semi-translucent bracts, monocarpic reproduction and continuous survival under stress. Here, we present genomic, transcriptomic and metabolomic analyses for one such plant, the Noble rhubarb (*Rheum nobile*). Comparative genomic analyses show that an expanded number of genes and retained genes from two recent whole-genome duplication events are both relevant to subnival adaptation of this species. Most photosynthesis genes are downregulated within bracts compared to within leaves, and indeed bracts exhibit a sharp reduction in photosynthetic pigments, indicating that the bracts no longer perform photosynthesis. Contrastingly, genes related to flavonol synthesis are upregulated, providing enhanced defense against UV irradiation damage. Additionally, anatomically abnormal mesophyll combined with the downregulation of genes related to mesophyll differentiation in bracts illustrates the innovation and specification of the glass-like bracts. We further detect substantial accumulation of antifreeze proteins (e.g. *AFP*s, *LEA*s) and various metabolites (e.g. Proline, Protective sugars, procyanidins) in over-wintering roots. These findings provide new insights into subnival adaptation and the evolution of glasshouse alpine plants.

## Abstract (web summary)

A chromosome-level genome assembly for the glasshouse plant *Rheum nobile* (noble rhubarb) combined with phylogenetic, transcriptomic, and metabolomic analyses provides insight into how alpine plants have adapted to growth in extreme environments.

## Introduction

**Fig. 1:**
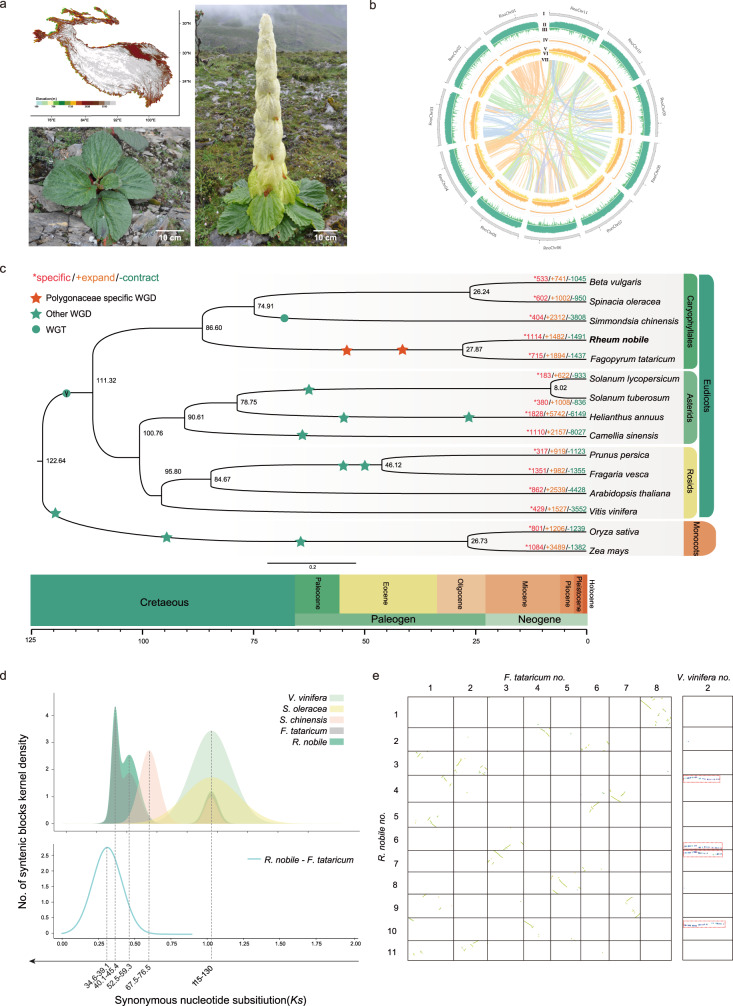
Typical habitat of noble rhubarb (*Rheum nobile*) and genome evolution analyses. **a** Altitude across the Qinghai-Tibet plateau (top left), a vegetative individual (bottom left) and a reproductive individual (right) of noble rhubarb. **b** Overview of the two Rheum genomes, the tracks (moving inward) show: (I) chromosomes; (II) gene numbers (0–100); (III) GC content (0.25–0.45); (IV) TE density (0–100%); (V) Gypsy density (0–90%); (VI) Copia density (0–60%); and (VII) identified syntenic blocks, calculated using 500 kb sliding windows. **c** Phylogenetic tree of 16 species and dating of WGD events. Gains and losses of gene families in sub-branches are highlighted in orange and green, respectively. Red, green and blue stars indicate Polygonaceae-specific WGDs, other reported WGDs, and whole-genome triplication events, respectively. **d** Distribution of average synonymous substitutions (*Ks*) between syntenic blocks after evolutionary rate correction. **e** Dotplots of interspecific syntenic blocks among *Vitis vinifera*, *Fagopyrum tataricum*, and *R. nobile*.

Subnival ecosystems, existing just below the permanent snowline in mountainous regions, are subject to high levels of irradiation, freezing temperatures and hypoxia throughout the year, is the most inhospitable climate zone at the highest altitude among terrestrial ecosystems, making it nearly lifeless for most higher animals and plants^1^. Such ecosystems are widely distributed in the high mountains of North America, Europe, and Asia (especially the Qinghai-Tibet Plateau [QTP])^2^ (Fig. 1a). All resident plant species in this zone face enormous challenges for survival. Thus, under selective pressure from a variety of stressful environmental factors, plants in this area have developed highly complex and elaborate adaptive evolutionary traits to ensure their survival and reproduction in extreme environments. For example, ‘glasshouse-like’, ‘cushion’ and ‘woolly’ phenotypes^3^. Of which, the “glasshouse structure” is one of textbook example for high-altitude adaptation in subnival belt, in which plants develop cream-colored translucent bract-like leaves during the reproductive phase. These bract-like leaves forming a glasshouse that plays a crucial role in ensuring successful reproduction and completing the plant’s life cycle. Alpine ‘glasshouse’ plants have been recorded in a range of plant families, for example the Ranunculaceae, Caryophyllaceae, Lamiaceae, Asteraceae and Polygonaceae.

As an iconic of alpine plant, Noble rhubarb (*Rheum nobile* Hook. F. et Thoms., Polygonaceae) were considered by Hooker and Fitch (1855) as “certainly the most striking of the many fine alpine plants of Sikkim”^4^. This giant herb occurs sparsely across subnival belts in the QTP at elevations between 4000 and 6000 m^5–7^ and at flowering possesses stacked layers of large and showy bracts that securely conceal the entire inflorescence, to produce a pagoda-shaped ‘glasshouse’ phenotype (Fig. 1b)^7,8^. These bracts aids reproduction in multiple ways^5,6,9–15^, which could increasing flower and fruit temperature within bracts and that higher by up to 10 and 8 °C, respectively^15^, than when bracts were removed or in the ambient conditions on sunny days, preventing pollen grains from being washed away by rain^9,15^, and intensity of ultraviolet-B (UV-B) radiation reaching flowers (or fruits) was decreased by 93–98% by bracts^5,11,15^, but there have been no previous attempts to investigate the genetic bases of subnival adaptation in these plants. Before flowering, plants develop for several years as a rosette during which they are subject to extremely low temperatures and strong winds, particularly during winter periods. Senescence of above ground parts is a response to such conditions^16^, but tissues below ground continue to survive rasing questions on how this is achieved and the mechanisms involved.

Here, we present a high-quality chromosome-scale genome assembly of the Noble rhubarb and detect the genetic legacy of two recent whole-genome duplication events that might have promoted its subnival adaption. In addition, we examine changes in gene expression and chemical production in its floral bracts and rosette roots based on transcriptomic and metabolomic analyses. Using these data we further investigate possible evolutionary adaptations underlying stress tolerance in this ‘glasshouse’ plant through detailed examination of expressed genes. Finally, subnival adaptation to extreme freezing environments during overwintering was examined using a multiomics approach. The availability of a reference genome sequence of *R. nobile*, together with information on the molecular basis and genetic mechanisms of adaptation to subnival environments, provide valuable resources for future evolutionary investigations of high alpine plants exposed to extreme ecological stress.

## Results

### Assembly and annotation of the high-quality genome

A high-quality chromosome-level genome sequence of *Rheum nobile* was produced using multiple technologies. In total, 217.64 Gb of Illumina reads (~135× depth), 93.26 Gb PacBio HiFi reads (~29× depth), and 189.62 Gb of high-throughput chromosome conformation capture (Hi-C) reads (~119× depth) were generated (Supplementary Table 1; depths based on estimated genome size, Supplementary Fig. 1a). Based on Hi-C integrated assembly of hifiasm, a primary assembly with long stretches of phased blocks and two phased contig graph were obtained, and the primary assembly were used in subsequent analysis. The sequence (1.57 Gb, N50 = 6.72 Mb) was very close to the estimated genome size (~1.59 Gb) based on k-mer distribution analysis (and Supplementary Table 2). After preliminary assembly, both purge_haplotigs and purge_dup were used to generate the final contig-level assembly to retain only one copy of each of the contigs from heterozygous regions. While the distribution of reads depth indicated there is no peak of heterozygous regions in purge_haplotype (Supplementary Fig. 1b), and purge_dup identified ~2 Mb junk region but not duplicate haplotig, these results also suggest high accuracy of the assembled genome. Then, the Hi-C dataset was further used to cluster and order contigs to generate a chromosome-level genome assembly. In total, 99.33% of assembled sequence could be anchored into 11 chromosomes (Fig. 1b, Supplementary Fig. 1c and Supplementary Table 3). To comprehensively assess the accuracy, continuity and completeness of our *R.nobile* genome, four analyses were used to evaluate the assembly quality. In total, the raw Illumina paired-end reads were mapped to the assembled genome with mapping rates and coverage of 99.92% and 99.67%, respectively (Supplementary Table 4). And the accuracy and integrenity of the assembly were evaluated by consensus quality value (QV score), k-mer completeness and long terminal repeat (LTR) Assembly Index (LAI) score. Of which, QV score and k-mer completeness was evaluated at 51.96 and 99.62% using Merqury, respectively (Supplementary Table 5) and LAI score of 25.08 were estimated (Supplementary Table 5), which suggested *R. nobile* assembly exceeds the minimum reference standard of 6.7.Q40 (e.g., >1.0 Mb contig) and 7.C.Q50 (e.g., chromosome-level, QV score>50, k-mer completeness > 95%, functional completeness>90%) set for eukaryotic species by the Earth BioGenome Project. Together, these two indices indicate high base accuracies and continuityfor our *R. nobile* genome. Moreover, Benchmarking Universal Single-Copy Orthologs (BUSCO) analysis indicated that 97.3% of the conserved single-copy eukaryotic genes were completely captured in the *R. tanguticum* genome assembly (Supplementary Table 6).

The majority (83.69%) of the genome consisted of transposable elements (TEs) distributed across all chromosomes (Fig. 1b and Supplementary Table 6). Long-terminal repeat elements (LTRs) dominated, accounting for 71.75% of all TE sequences (Supplementary Table 6). *Copia* and *Gypsy* were the two major families of LTRs, comprising 0.22 Gb and 0.60 Gb of the genome, respectively. Both LTR families showed a sudden increase in frequency in the genome ~3 million years ago (Mya), with *Copia* subsequently undergoing a rapid decrease while *Gypsy* families remained relatively stable in frequency (Supplementary Fig. 1d). We predicted a total of 37,770 protein-coding genes in the genome with complete BUSCO values of 95.6% (Supplementary Tables 7 and 8). Average protein-coding gene length was 4314 bp and mean exon number ~5 (Supplementary Table 7). Approximately 90.7% of genes were functionally annotated through Blast searches of five functional databases (Supplementary Table 9). In addition, 2341 transcription factors and 10,947 non-coding RNAs were identified (Supplementary Table 10, 11).

### Genome evolution

Gene sequences from 15 species (*R. nobile* and four other, four asterids, four rosids and two monocots [rice and maize]) were clustered and assigned to 46,684 gene families. Of these, 1098 single-copy gene families were determined and used in phylogenetic analysis. Both coalescent-based and concatenation-based phylogenies with high support (posterior probability values of 1 and bootstrap values of 100) showed consistent topology with those previously report (Fig. 1c and Supplementary Fig. 2)^17–20^. Noble rhubarb was estimated to have diverged from Tartary buckwheat (*Fagopyrum tataricum*, Polygonaceae) ~ 27.87 million years ago (Mya) (Fig. 1c). Dating further showed that Polygonaceae species diverged from Amaranthaceae (including beet [*Beta vulgaris*] and spinach [*Spinacia oleracea*]) and Simmondsiaceae (including jojoba [*Simmondsia chinensis*]) ~86.60 Mya, and that Caryophyllales diverged from asterids and rosids ~111.32 Mya (Fig. 1c).

We identified 1482 expanded gene families in *R. nobile* (Fig. 1c) with functions mainly associated with DNA damage repair, possibly of adaptive significance in subnival areas subject to high UV radiation (Supplementary Data 1 available on Figshare; 10.6084/m9.figshare.19662933). Gene functions associated with leaf morphogenesis and cotyledon morphogenesis were also enriched (Supplementary Data 1 available on Figshare; 10.6084/m9.figshare.19662933) and may combine in the formation of the ‘glass-house’-like plant organ. In addition, 1,114 unique gene families with functional enriched categories were mainly implicated in plant defense (Supplementary Data 1 available on Figshare; 10.6084/m9.figshare.19662933).

The distribution of average synonymous substitution rates (*Ks*) of intra-genomic collinear gene blocks indicated that two polyploidization events occurred in the evolutionary histories of both *R. nobile* and Tartary buckwheat (Fig. 1d and Supplementary Data 2 available on Figshare; 10.6084/m9.figshare.19662933) in addition to the γ event (whole-genome triplication) shared by all core eudicots^21,22^. Analyses of synteny depths supported this (Fig. 1e and Supplementary Fig. 3a). Previously, it was thought that only one additional WGD event had occurred in Tartary buckwheat^17^. Because beet and spinach genomes have undergone complex chromosome rearrangement events during their evolution^23^, we selected the genome of *Vitis vinifera* as a reference for further analyses. This genome underwent only the γ event with fewer subsequent chromosomal rearrangements. We obtained synteny depth ratios of 1:4 between *V. vinifera* and both Polygonaceae species (Fig. 1e), indicating that the two recent shared polyploidization events in these species were both whole-genome duplications (WGDs). The two recent WGDs among Polygonaceae species received further support from phylogenetic analyses of all identified collinear genes (Supplementary Fig. 3b), and after correction for evolutionary rates^24^ were estimated to have occurred 52.35-59.32 and 40.19-45.43 Mya, respectively, that is before divergence of *R. nobile* from Tartary buckwheat (Fig. 1c, d). Notably, compared to other four Caryophyllales species that used in this study, including the Tartary buckwheat which shared the same polyploid history, the rhubarb genome exhibited a higher retention of genes originating from WGD events (Supplementary Fig. 4). And these genes were enriched (*inter alia*) in the GO terms that related to freezing stress response (GO:0050826), and some GO terms related to osmotic stress response (e.g.: GO:0047484, GO:1901002, GO:1901000, GO:1902074), which may be related to its cold stress response, as freezing stress often leads to cellular dehydration and subsequent osmotic stress. And some GO terms that related to hypoxia response (e.g.: GO:0036293, GO:0001666, GO:0070482) were only enriched in the *R. nobile* genome. Moreover, numerous WGD originated genes were also enriched in GO terms that related flavonoid biosynthetic process (e.g.: GO:0009963, GO:0009699, GO:0009813, GO:0051555, GO:0009962), which also is an important class of secondary metabolites play a role in plant defense to multiple stressors, indicating that *R. nobile* may have partially acquired the ability to adapt to harsh environments through specific retention of WGD originated genes (Supplementary Data 3 available on Figshare; 10.6084/m9.figshare.19662933).

### Gene expressions and metabolites accumulations of the innovative ‘glasshouse’-like bracts

**Fig. 2:**
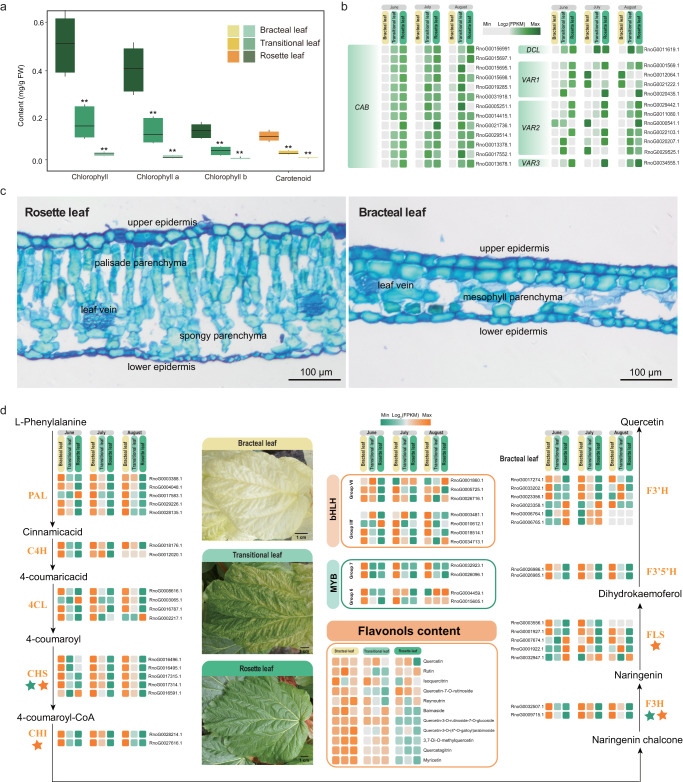
Innovation of glasshouse-like bracts. **a** Chlorophyll (green) and carotenoid (orange) contents in three types of leaves (bract, transitional leaf and rosette) of *R. nobile*. Three independent biological replicates were used to estimate means (*n* = 3 biologically independent samples) and standard deviations (SD) in each case. For each photosynthetic pigment, *t* tests were applied to test the significance of differences in contents between bracts and leaves (rosette and transitional). Double asterisks indicate *p* < 0.01. **b** Heatmap of expression of genes involved in chloroplast and mesophyll development in leaves. Gene expression profiles (in fragments per kilobase of exon per million mapped fragments) in the three types of leaves at three time points in the growth season (here June, July and August) are shown from left to right. Colors from gray to green indicate gene expression levels from low to high. Gene names are shown beside each heatmap. *CAB*, *CHLOROPHYLL A/B BINDING PROTEIN*; *DCL*, *DEFECTIVE CHLOROPLASTS AND LEAVES*; *VAR*1, *VARIEGATED 1*; *VAR2*, *VARIEGATED 2*; *VAR3*, *VARIEGATED 3*. **c** Cross-sections of rosette leaf (leaf) and bracteal leaf (right) of *R. nobile* that stained with 0.1% toluidine blue. Bar = 100 μm. **d** Expression patterns of genes associated with flavonoid biosynthesis and accumulation in indicated tissues, obtained using same tissue panel as in (**b**), with low to high expression/content indicated by colors from green to orange. PAL phenylalanine ammonia-lyase, C4H cinnamate-4-hydroxylase, 4CL 4-coumarate CoA ligase 4, CHS chalcone synthase, CHI chalcone isomerase, F3H flavanone 3-hydroxylase, FLS flavonol synthase, F3′5′H flavonoid 3′,5′-hydroxylase; F3′H flavonoid 3′-hydroxylase, MYB myeloblastosis transcription factor, bHLH basic helix–loop–helix transcription factor.

‘Glasshouse’ plants are characterized by large and showy semi-translucent bracts concealing a compound raceme. To explore the genetic basis of bract formation, we initially compared the regulatory mechanisms of gene expression underlying structural differences between bracts and leaves. Fewer chloroplasts with severe developmental defects are also evident in bracts^6^. For transcriptome analyses we collected 27 samples of three tissues (bracts, transitional leaves and rosette leaves) at three different times throughout the growing season (June, July and August) (Fig. 2, Supplementary Fig. 5 and Supplementary Table 12). We detected 5142 genes that were expressed significantly less strongly in bracts than in transitional leaves or rosette leaves (Supplementary Fig. 5). The functions of these genes were mainly associated with photosynthesis, such as ‘photosynthetic, light harvesting’, ‘photosynthetic electron transport chain’, ‘photosynthesis’, ‘chlorophyll biosynthetic process’ and ‘carotenoid biosynthesis’ (Supplementary Data 4 available on Figshare; 10.6084/m9.figshare.19662933) with genes related to both chlorophyll and carotenoid biosynthesis also downregulated in bracts (Supplementary Figs. 6 and 7). Bracts were found to have 87.27 and 95.63% lower chlorophyll a/b contents than transitional and rosette leaves, and exhibited similar reductions for carotenoid contents (ca. 88.78 and 93.52%, respectively) (Fig. 2a). Key genes involved in chloroplast and mesophyll development in leaves were weakly expressed in bracts (Fig. 2b, Supplementary Fig. 8), for example, multiple copies of *CHLOROPHYLL A/B BINDING PROTEIN* (*CAB*)^25,26^. Also, most *VARIEGATED* (*VAR*) genes were expressed significantly less strongly in bracts (Fig. 2b). *VAR1*^27,28^ and *VAR2*^27,29^ play important roles in photoprotection and development of thylakoid membranes, while absence of *VAR3*^30^, which is required for chloroplast and palisade cell development, results in lower levels of chlorophylls and carotenoids. It was further evident that *DEFECTIVE CHLOROPLASTS AND LEAVES* (*DCL*)^31^ genes involved in palisade morphogenesis were expressed extremely weakly in bracts (Fig. 2b). This was of interest as cross-sections of rosette leaf (left) and bracteal leaf (right) mesophyll parenchyma showed that bracts comprised only 3–4 layers of cells and lack a distinct palisade and spongy parenchyma present in rosette leaves (Fig. 2c).

The distinctive structure of bracts enables high light transmittance, thus warming the reproductive organs inside the ‘glasshouse’. In addition, the high content of UV-absorbing compounds (e.g., flavonols and related phenylpropanoid derivatives) protect reproductive organs from the damaging effects of UV irradiation^11^. We quantified flavonoids based on our metabolomic data, and detected substantially higher levels of flavonols, especially quercetin and myricetin derivatives (e.g., quercetin-3-O-rutinoside-7-O-glucoside, 3,7-di-O-methylquercetin, myricetin and myricetin-3-O-rutinoside) in bracts than in leaves (Fig. 2d Supplementary Data 5 available on Figshare; 10.6084/m9.figshare.19662933). Transcriptome data further indicated that 4863 genes associated with ‘Flavonoid biosynthesis’ and ‘Phenylpropanoid biosynthetic process’ (Supplementary Data 6 available on Figshare; 10.6084/m9.figshare.19662933) were more highly expressed in bracts than transitional or rosette leaves. Moreover, all genes encoding the nine enzymes involved in the flavonoid biosynthetic pathway were more strongly expressed in bracts than in leaves at each time of analysis (Fig. 2d). This was also the case for transcription factors (including both *bHLHs* (Groups VII and IIIf) and *MYBs* (Groups 6 and 7)) related to regulation of flavonoid biosynthesis (Fig. 2d)^32–36^. And 5 mature miRNAs were also found showed high similarity (>85%) with miRNAs involved in regulating flavonoid biosynthesis in *Arabidopsis* (e.g.: miR166, miR177 and miR396) may also participated in the flavonoid accumulation in *R. nobile* (Supplementary Table 13). Taken together, these results suggest continuously strong biosynthesis and concentration of flavonoids in bracts.

Approximately 26.5% (1288 genes) of upregulated genes in bracts are remnants of whole-genome duplications and linked to ecological functions of bracts. These genes were found not only enriched in auxin efflux, plant epidermis and stomatal complex development, flower development and flavonoid biosynthesis, but also enriched in response to blue light, glutathione metabolic process and shoot system morphogenesis (Supplementary Data 7 available on Figshare; 10.6084/m9.figshare.19662933). These results indicate that WGD contributed to bract development in *R. nobile*.

### Overwintering of the perennial root

**Fig. 3:**
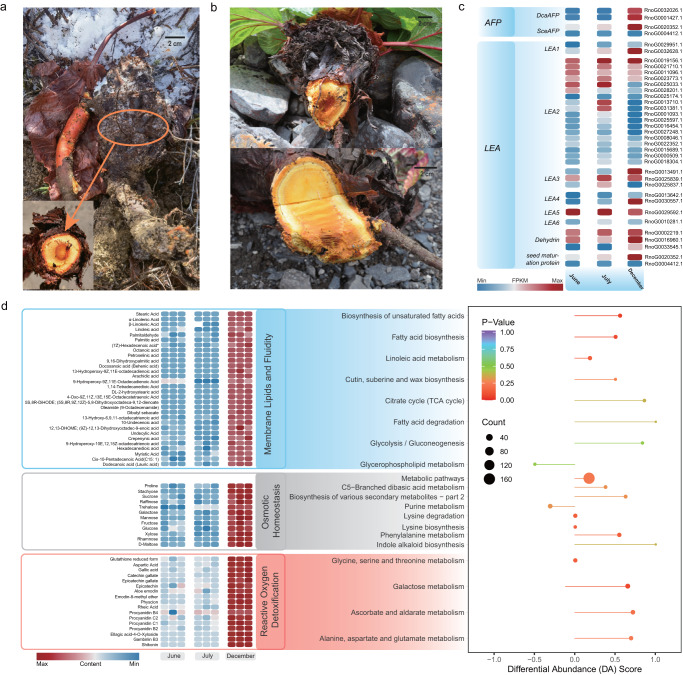
Adaptive genetic basis of perennial root of *R. nobile* to overwinter. **a**, **b** Images illustrating the morphology of *R. nobile* in its natural habitat during winter and summer, respectively, with zoomed lateral section of thick taproots sampled for sequencing. **c** Expression profiles (in fragments per kb of exon per million mapped fragments) of two genes encoding antifreeze proteins in roots collected at three time points (June, July and December). Low to high expression is indicated by colors from blue to red. **d** Metabolomic regulation of responses to the extremely low temperatures during wintertime in the subnival belt. Levels of three classes of metabolites in roots in indicated months are presented as heatmaps (left). Differential abundance scores (DA Score) calculated for corresponding KEGG pathways are presented in the lollipop chart (right). Horizontal coordinates of DA Scores reflect overall changes in levels of all metabolites in a given metabolic pathway. Scores of 1 and −1 indicate trends of up- and down-regulation of all identified metabolites in the pathway, respectively. The length of the line segment indicates the absolute value of DA Score, and the size of the dots at the end points of each line segment indicates the number of differential metabolites in the pathway. Low to high expression is indicated by colors from blue to red.

Extremely low temperatures during wintertime in subnival regions pose severe challenges for perennial plants. Overwintering without injury may require multiple adaptations, involving physiological and biochemical modifications, alterations of gene expression, and changes in concentration of specific proteins and metabolites^37–40^. To assess such changes in roots of *R. nobile*, root samples collected during the growing season (June and July) and wintertime (December) were subjected to transcriptome and metabolic analyses (Fig. 3a, b, Supplementary Fig. 9 and Supplementary Table 14). Transcription was downregulated during winter of genes involved in regulation of auxin-mediated signaling pathways, plant hormone signal transduction and several biogenesis and developmental processes (Supplementary Data 8 available on Figshare; 10.6084/m9.figshare.19662933). Ribosomal function and biogenesis, however, were maintained in a state of transcriptional readiness during this period to initiate protein synthesis, with transcription of genes involved in these pathways upregulated (Supplementary Data 9 available on Figshare; 10.6084/m9.figshare.19662933). Thus, ribosome biogenesis and recycling of cytosolic ribosomes^41,42^ continued even at very low temperatures. In addition, the expression of two gene families encoding antifreeze proteins (*AFP*)^43–47^, and 11 *Late Embryogenesis Abundant* (*LEA*) genes^48^ involved in cold tolerance of plants, were upregulated in roots during the winter. These changes are likely to increase low temperature tolerance by reducing freezing points of all active systems (Fig. 3c).

Metabolomic analyses provided further evidence for root overwintering ability in extremely cold subnival habitats (Fig. 3d and Supplementary Fig. 9). We identified 1,091 metabolites in root tissues relevant to survival in freezing conditions (Fig. 3d and Supplementary Data 10 available on Figshare; 10.6084/m9.figshare.19662933) with multiple KEGG categories related to biosynthesis of unsaturated fatty acid enriched in overwintering roots (e.g., ‘Biosynthesis of unsaturated fatty acids’, ‘Linolenic acid metabolism’ and ‘Fatty acid biosynthesis’) (Fig. 3d). Similarly, diverse compatible osmolytes were significantly more abundant in winter roots, e.g., proline, which is required to maintain cytosolic acidity and membrane integrity^37^, and various soluble sugars (sucrose, raffinose, fructose, trehalose, etc.) that can lower a cell’s osmotic potential and stabilize cellular and protein structures (Fig. 3d)^37^. These modifications likely offset the adverse effects of low temperatures on cell membrane properties and functions caused by reduced membrane fluidity^37,49,50^. Freezing temperatures can also induce severe oxidative stress through generation of reactive oxygen species (ROS). Excessive accumulation of ROS leads to cellular injury, and ultimately death of plants by damaging the photosystem II reaction center and membrane lipids^37^. Multiple antioxidant metabolites (e.g., reduced glutathione, procyanidins, gallic acid and epicatechin gallate) were significantly more abundant in winter roots, as were several categories of metabolites related to the Ascorbate-Glutathione (AsA-GSH) cycle (‘Ascorbate and aldarate metabolism’, ‘Alanine, aspartate and glutamate metabolism’, ‘Glycine, serine and threonine metabolism’, etc.), which play key roles in ROS scavenging (Fig. 3d)^51,52^. These results suggest, therefore, that improved membrane stability, osmolarity, and resistance to ROS during periods of very low temperatures have developed in *R. nobile* through adjustment of physiological pathways and modification of metabolic profiles.

Notwithstanding the above, comparisons show a major distinction exists between transcriptional and metabolic responses of *R. nobile* to freezing stress, as overwintering roots had massive levels of associated metabolites but correlated pathways were retained in relatively quiescent transcriptional states. This may arise from a time lag between transcript and metabolite accumulation. It is essential for plants to accumulate sufficient relevant metabolites to survive in sub-zero temperatures during winter, while correlated transcripts may be largely generated during autumn^37,53,54^.

## Discussion

Driven by extreme selection pressures, alpine subnival plants have developed diverse specialized adaptive traits and evolutionary strategies to survive and reproduce in high stress environments^3,55^. To explore the underlying genetic basis of some of these adaptations, we generated a chromosome-level genome assembly of the ‘glasshouse’ *R. nobile*, an iconic subnival species that occurs at elevations between 4000 m and 6000 m in the QTP. The *R. nobile* genome was ~1.57 Gb in size, and 99.33% of the sequences were assigned to 11 pseudochromosomes based on Hi-C auxiliary assembly. The higher scaffolding rate of contigs and all four indices (mapping rate, BUSCO, QV and LAI) highlighted the higher quality of our *R. nobile* genome assembly than recently published *R. nobile* assembly^56^ (Supplementary Table 15). To obtain highly reliable gene models, initial gene set were further filtered and a gene set containing 37,770 genes was finally obtained which number close to its close relative buckwheat, and with high functional completeness (>90%). Thus, a more accurate picture of gene order and genome structure and a valuable genetic resource of alpine plant were provided for future study related to adaptive evolution of plants.

**Fig. 4:**
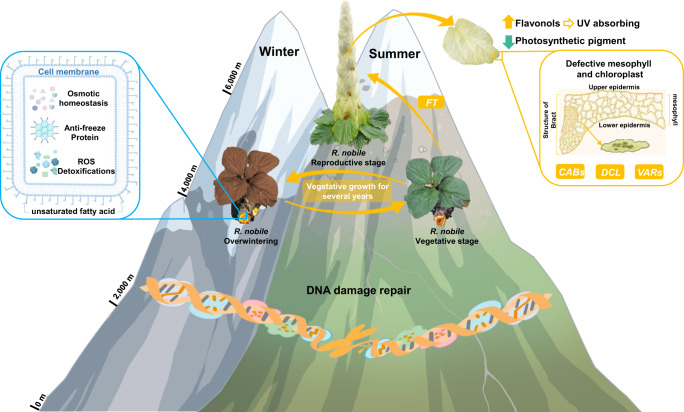
A scheme of subnival adaptation in *R. nobile*. Indicated are: expanded gene families and WGD-retained genes for reducing DNA damage caused by strong UV radiation; accumulation of anti-freeze proteins and metabolites facilitating overwintering.

In contrast to previous indications of only one recent WGD event occurring Polygonaceae species (*Fagopyrum tataricum*) or failure distinguishing for WGD or WGT in *R. nobile* assembly which published recently, we revealed two recent WGD events shared by Polygonaceae species, dated to ~40.2-45.4 and ~52.5-59.3 Mya, respectively. It was because when multiple round WGD events occurred in a very short time, the *Ks* distribution or *Ks* peak fitting may induce errors as only give one peak (corresponding one WGD event). This is a common phenomenon and the errors about the WGDs in *Simmondsia chinensis*, *Carthamus tinctorius* and *Olea europaea* had been reported^57^. In our study, we identified two adjacent *Ks* peaks in a very short time (*Ks* range: 0.3–0.5) (Fig. 1d), suggesting two WGD events occurred in *R. nobile* and *F. tataricum*. Our synteny gene dotplot analyses between the two Polygonaceae species and *V. vinifera* (Fig. 1e and Supplementary Fig. 3a) showed clear evidences of synteny depth ratio of 1:4 between *V. vinifera* and the two Polygonaceae species, which means every chromosome of *V. vinifera* could be find four copies in the two Polygonaceae species and also could be another evidence for two rounds of WGDs. Also, the synteny genes between the species were extracted to construct the phylogenetic tree, and most phylogenetic topology of gene trees supporting the shared WGD events between the two species(Supplementary Fig. 3b). Moreover, WGD is considered to contribute greatly to adaptive evolution because it provides additional genetic materials for divergent selection to act on as a consequence of neofunctionalization and increased gene product dosage^58,59^. Notably in *R. nobile*, a high retention was evident of duplicated genes that were annotated likely to promote subnival adaptation. Thus, responses to hypoxia and osmotic stress, and cellular responses to lipid were significantly enriched, which is highly suggestive of the important role of WGD events in adaption to extremely freezing temperatures. In addition, expanded gene families were detected that were mainly related to DNA damage repair and leaf development (Fig. 4), possibly enabling adaptation to high UV radiation stress at subnival elevations and formation of the ‘glasshouse’ reproductive structure.

The innovative development of translucent cream-colored bracts which combine to conceal the inflorescence and produce a pagoda-shaped ‘glasshouse’ phenotype^4,7,8^, likely insures successful flowering and seed development of this giant monocarpic perennial^5,6,9–12,14,15,60^. Tissue-specific genetic modifications affecting multiple regulatory pathways are likely associated with the adaptability of this trait. In support, we detected significant downregulation in the bracts of key genes involved in chloroplast and mesophyll development (*CAB*s), palisade morphogenesis (*DCL*), and photoreception and thylakoid membrane development (*VAR1*, *VAR2* and *VAR3*). Anatomical abnormality of mesophyll and dramatic decrease of content for both of two photosynthetic pigments (chlorophylls and carotenoids) in bracts further verified the results of transcriptomics, and all above completely reveal specialization of adaptive function of bracts. On the other hand, avoidance of high UV irradiation is likely to result from the significantly enhanced activities detected for enzymes involved in the flavonoid biosynthesis, and instead of only a handful of genes in previous studies, we found whole pathway of flavonoid biosynthesis, even related transcription factors (*R2R3-MYB* and *bHLH*) which were confrimed regulate the accumulation flavonoid all exhibit tissue-specific expression. Moreover, retention of a high proportion of duplicated genes up-regulated in bracts and enrichment of genes related to flavonoid biosynthesis suggest that WGD substantially contributed to the innovative function of bracts. In combination, these changes are likely to have increased the translucence of bracts while maintaining a robust tolerance to strong UV exposure.

Overwintering vegetative growth is also critical for this monocarpic perennial to survive subnival extremes over multiple years. However, research on *R. nobile* is mostly focused on its ‘glasshouse’ reproductive structure, and there is very little research related to the freezing tolerance mechanisms. In addition to the genetic changes involved in the production of the ‘glasshouse’ bract, several changes in gene expression and metabolic accumulation were detected firstly in the roots of *R. nobile* that are likely to improve their survival during freezing winters. These included alterations to expression of cold tolerance genes leading to accumulation of several protective proteins including antifreeze proteins (*AFP*s) and late embryogenesis abundance (*LEA*s), plus changes in concentrations of proteins and metabolites (unsaturated fatty acids, soluble sugars, proline, and antioxidants) enormously enhance accomplishment of basic requirements of carbon source, energy, membrane stability, osmolarity, and resistance against ROS produced during freezing stress (Fig. 4).

In combination, our findings have deepened considerably our current understanding of the likely genetic changes that have occurred to enable ‘glasshouse-like’ plants to survive and reproduce under some of the most hostile conditions that plants grow in.

## Materials and methods

### Genome sample collection and sequencing

Fresh leaf tissue was sampled from a wild individual of *R. nobile* growing on Mount Segrila, Tibet, China (29°37′5.87″ N, 94°38′59.84″E, 4,628 m) and immediately stored in liquid nitrogen before sending to Grandomics (Wuhan, China) for genomic sequencing. High molecular weight genomic DNA was prepared by the CTAB method then purified with a QIAGEN^®^ Genomic kit (Cat. No. 13343, QIAGEN). To obtain Illumina short reads, DNA libraries with 500 bp inserts were constructed and sequenced using an Illumina HiSeq 4000 platform. In addition, high-molecular-weight DNA was prepared and used to construct PacBio SMRTbell libraries using a SMRTbell Express Template Prep Kit 2.0, following the manufacturers’ recommended protocols. The SMRTbell libraries were sequenced using a PacBio Sequel II system and consensus (HiFi) reads were generated using ccs software (https://github.com/pacificbiosciences/unanimity). Hi-C (high-throughput chromosome conformation capture) sequencing was performed as follows: sampled DNA was cross-linked with 1% formaldehyde to capture interacting DNA segments, chromatin was digested with the Dpn II restriction enzyme, and libraries were constructed and sequenced using the Illumina HiSeq 4000 platform.

### Genome size estimation and assembly

Before estimating genome sizes, short Illumina reads were filtered using fastp (v.0.20.0)^61^ with default parameters. Clean reads were then used to generate *K*-mer (21 bp) frequencies by Jellyfish (v.2.2.10)^62^, and the resulting histogram was exported into GenomeScope (v.1.0.0)^63^. Hifiasm (0.15.3-r339)^64^ was used for de novo assembly of *R. nobile*’s genome with default parameters with HiFi sequencing data. A subprogram of Purge Haplotigs (v.1.1.2)^65^ and purge_dup (v.1.2.5)^66^ was used to identify uncollapsed duplications. The quality of the assembly was comprehensively sessed by using four methods: (i) Mapping the Illumina paired-end reads to our final assembly shows high completeness of the genome when high mapping rates are obtained; (ii) BUSCO (Benchmarking Universal Single-Copy Orthologs) (v.5.2.1)^67^ was used with the embryophyta_odb10 database and a high percent of complete BUSCOs also indicates high completeness of the genome; (iii) the consensus quality value (QV score) evaluated using Merqury (v.1.3)^68^ indicates high base accuracies of the genome with a high QV score; (iv) the long terminal repeat (LTR) Assembly Index (LAI) evaluated using LTR_retriver (v2.8.5)^69^ serves as the gold standard for genome benchmarking when LAI > 20. Clean Hi-C data were mapped to contig sequences by BWA-MEM (0.7.10-r789)^70^ and valid interaction pairs were extracted. Based on those chromatin interactions, 3D-DNA (v.180922)^71^ was employed to cluster, order, and orient the contigs into pseudo-chromosomes. Juicebox^72^ was used to visualize the chromatin interactions among the assembled pseudo-chromosomes, and then we manually corrected and validated the obvious Hi-C assembly errors to generate the final chromosome assembly.

### Repeat element identification and gene prediction

RepeatMasker (v.4.1.0)^73^ and RepeatProteinMasker (v.4.1.0)^73^ were used to identify repetitive elements in noble rhubarb genome based on homology alignments between our assembly sequences and Repbase (v.16.10). We then applied the de novo approach to improve the sensitivity of our repeat identification. Briefly, RepeatModeler (v.2.0)^74^ and LTR_Finder (v.1.06)^75^ were used to construct a repeat library, then RepeatMasker^73^ was employed to generate de novo predictions.

A combination of transcriptome-based, homology-based, and de novo approaches was used to accurately predict high-quality protein-coding genes. To predict genes ab initio, Augustus (v.3.2.3)^76^, GenScan (v.2.0)^77^, and GlimmerHMM (v.3.0.4)^78^ were employed with the *Arabidopsis thaliana* training set. GeMoMa (v.1.7.1)^79^ was used for homology-based prediction, together with protein sequences from *A. thaliana*^80^, *Beta vulgaris*^19^, *Fagopyrum tataricum*^17^, *Prunus persica*^81^, and *Spinacia oleracea*^82^ (Supplementary Table 16). For transcriptome-based prediction, the fresh leaf tissues that from the genome sequencing individual were collected for the annotation, then the de novo transcriptome assemblies of the leaf tissues were aligned to the genomes to resolve gene structures using PASA. EVidenceModeler (EVM, v.1.1.1)^83^ was used to generate consensus sets of gene models obtained using the three approaches (transcriptome-based, homology-based, and de novo approaches). To obtain highly reliable gene models, we fitted genes that were only supported by transcriptome-based prediction and with just one exon, and also those only supported by the ab initio process with less than three exons. Protein-coding genes were functionally annotated by BLASTP (v.2.7.1+)^84^ (E-value < 1 × 10^−5^) searches against SwissProt and TrEMBL databases. InterProScan (v.5.28)^85^ was used to annotate protein domains by searching the InterPro databases. Gene Ontology (GO) terms for each gene were obtained from the corresponding InterProScan results. Pathways in which each gene might be involved were assigned by BLAST searches against the Kyoto Encyclopedia of Genes and Genomes (KEGG) database^86^. Transcription factors in noble rhebarb were detected using iTAK^87^. Non-coding RNAs (ncRNAs) were annotated using cmscan from INFERNAL (v1.1.2) (http://eddylab.org/infernal). The mature miRNAs were further identified using miRPara (v6.0)^88^ based on the results of cmscan subprogram, and the target genes of the mature miRNAs were predicted by psRobot (v1.2)^89^ with default parameters.

### Phylogenetic analysis and expansion/contraction of gene families

To investigate the evolutionary trajectories of *R. nobile*, we selected 14 other species for phylogenetic analysis (Supplementary Table 16): *Arabidopsis thaliana*^80^, *Beta vulgaris*^19^, *Camellia sinensis*^90^, *Fragaria vesca*^91^, *Fagopyrum tataricum*^17^, *Helianthus annuus*^92^, *Oryza sativa*^93^, *Prunus persica*^81^, *Simmondsia chinensis*^20^, *Solanum lycopersicum*^94^, *Solanum oleracea*^82^, *Solanum tuberosum*^95^, *Vitis vinifera*^23^, and *Zea mays*^96^. An all-vs-all BLASTP^84^ search (E-value cutoff: 1 × 10^−5^) was first employed to generate similarity information for all genes. Next, we identified high-quality single-copy genes by applying OrthoMCL (v. 2.0.9–4)^97^, and constructed a concatenation tree and clusters of gene trees using IQ-TREE (v. 2.0.3-h176a8bc_0, with ‘-m MFP –bb 1000’ settings)^98^ and a coalescent tree by ASTRAL (v.5.7.8)^99^. We further estimated divergence times between species with MCMCtree (v.4.8)^100^ of the PAML package (v.4.8)^101^. Divergence times between *A. thaliana* and *V. vinifera* (115-130 Mya) and *B. vulgaris* and *S. oleracea* (22-30 Mya) acquired from TimeTree (http://www.timetree.org/)^102^ were used as calibration points. Gene family expansions and contractions were further estimated by CAFÉ (v.4.2)^103^ using the gene cluster information and estimated time tree. The parameter λ was estimated along each branch with the random model, and gene families were classified into four types: expanded, contracted, unique, or unchanged.

### Detection of WGD events

Two Polygonaceae species (*Rheum nobile* and *Fagopyrum tataricum*), together with *Simmondsia chinensis* and *B. vulgaris* of the Caryophyllales, were used for WGD analyses. Synteny blocks and collinear genes were identified by WGDI (v.0.5.3)^104^ with ‘-icl’ within each species and between Polygonaceae species. Numbers of synonymous substitutions per synonymous site (*Ks*) between collinear genes were also estimated by ‘-ks’ in WGDI, and a median Ks value was selected to represent each syntenic block, with Ks peak fitting also performed by WGDI with ‘-pf’. Dot plots of collinear genes and synteny blocks were used to obtain syntenic ratios between the species to confirm the polyploidy level of each species. We further used the collinear genes for phylogenomic analyses to check if detected WGDs occurred independently in the histories of *R. nobile* and *F. tataricum*. Collinear genes between pairs of these two Polygonaceae species were extracted by WGDI with ‘-at’, and IQ-TREE (v. 2.0.3-h176a8bc_0)^98^ was used to construct gene trees. For each gene tree, we randomly rooted a collinear gene from one species then checked whether the retained collinear genes from that species could be clustered as a monophyletic clade to support the hypothesis that independent WGDs occurred in these two species’ histories. Finally, we calculated the frequency of gene trees that supported independent WGDs for each species.

### Estimation of TE insertion times

The 5ʹ-LTR is usually identical to the 3′-LTR when a retrotransposon is inserted. Thus, in this analysis only LTR sequences identified with complete 5ʹ-LTR and 3ʹ-LTR were used. Each of the 5ʹ-LTR flanking sequences and 3′-flanking sequences was aligned by MUSCLE (v.3.8.31)^105^ with default parameters and evolutionary distances of aligned sequences were calculated by disMat (EMBOSS: v.6.6.0.0, with parameters -nucmethod 2)^106^. The mutation rates (per base per year) were claculted by r8s^107^ (1.6 × 10^−9^ for *R. nobile*), then the insertion times were further calculated using the formula: T = K/2r (divergence between LTRs/substitution per site per year).

### Chlorophyll and carotenoid concentrations

Total chlorophyll and carotenoids were extracted from bracts, transitional leaves and rosette leaves using the dimethylsulfoxide (DMSO) method^108^. The absorbance of each resulting extract (with three technical replicates of each of three biological replicates) was spectrophotometrically measured at 647, 663, 652 and 440 nm wavelengths. Total chlorophyll, chlorophyll a/b and carotenoid contents were calculated^109^.

### Cross-sectioning and histological staining

The rosette leavea and bracteal leaves were cut into 0.5–1 cm^2^ fragments, fixed in FAA solution, and embedded in paraffin. A rotary microtome (RM2235, Leica) was used to section the embedded fragments to a thickness of 10 μm. The sections were stained with 0.1% toluidine blue and observed using an optical microscope (Zeiss, Germany).

### Transcriptome sequencing and analysis

For transcriptome analysis, bract, transitional leaf, rosette leaf, and root samples of *R. nobile* were collected, with three replicates per tissue, on 19 August 2020 and 5 June, 6 June, 6 July, and 4 December 2021. Information on sample collection procedure for each tissue type is given in Supplementary Tables 12 and 14. Total RNA extraction, library construction and sequencing were performed by BGI-Shenzhen Company (Wuhan, China) using a MGI2000 platform and 2 × 150 bp pair-end model. After filtering low quality reads by fastp, clean reads were mapped to the *R. nobile* genome assembly using HISAT2 (v.2.2.1)^110^. StringTie (v.2.1.2)^111^ was used to predict new transcripts, which were combined with gene annotations to obtain a final transcriptome set. DEseq2 (v.1.22.2)^112^ was used to identify differentially expressed genes (DEGs), defined as those with |log2(fold change) |>1 and FDR significance score (*P*_adj_) < 0.05. DEGs were subjected to KEGG and GO enrichment analysis using clusterProfiler^113^.

### Metabolite profiling and analysis

For metabolomic analysis, bract, transitional leaf and rosette leaf, and root samples of *R. nobile* were collected, with three replicates per tissue (leaves and roots on 6 July 2021, and overwintering on 4 December 2021). Information on sample collection procedure for each tissue type is given in Supplementary Tables 12 and 14. Total metabolite extraction and analysis were performed by Mateware Company (Wuhan, China) using a SHIMADZU Nexera X2 ultra-high pressure liquid chromatography system (www.shimadzu.com.cn) coupled to an Applied Biosystems 4500 Q TRAP electrospray ionization-tandem mass spectrometry system (www.appliedbiosystems.com.cn/). Metabolite data were log2-transformed for statistical analysis to improve normalization. Metabolites from 18 samples were used for Hierarchical Clustering Analysis (HCA), Principal Component Analysis (PCA), and Orthogonal Partial Least Squares-Discriminant Analysis (OPLS-DA) using R software to study accession-specific metabolite accumulation, with significant p and fold change thresholds set to 0.05 and 2.0, respectively. Venn diagrams were used to illustrate the distribution of metabolites, and the KEGG database was used to identify metabolites that were differentially expressed (with a significance threshold of *p* < 0.01) in (i) bracts compared to transitional leaf and rosette leaves, and (ii) between roots in summer and winter.

### Analysis of photosynthetic pigment biosynthesis genes

Chlorophyll biosynthesis involves 15 catalytic steps from L-glutamyl-tRNA to chlorophyll *a* and chlorophyll *b*^114^. Genes encoding these 15 enzymes reportedly play key roles in changes in chlorophyll content in *A. thaliana* (https://www.arabidopsis.org/). Similarly, carotenoid synthesis involves 15 steps from geranylgeranyl pyrophosphate (GGPP) and 13 enzymatic reactions (KEGG: map00906), with corresponding genes being key determinants of changes in carotene content in *A. thaliana*. Thus, proteins encoded by these *Arabidopsis* genes were used to search for homologs in the predicted proteome of *R. nobile* using BLASTP^84^ with an e-value of 1e-5, >40% identity value and >40% coverage. In addition, domains of these *A. thaliana* genes were identified with hmmscan^115^, then applied in further searches against the *R. nobile* proteome using hmmsearch^115^.

### Genes related to chloroplast and mesophyll development

Genes reportedly involved in regulation of chloroplast development and mesophyll differentiation were downloaded. Nine (*ARC*, *CAB*, *CUE1*, *HB1*, *PHTO2*, *SCA3*, *VAR1*, *VAR2*, and *VAR3*) were retrieved from the *A. thaliana* genome and one (*DCL*) from the tomato genome. These genes were used as queries for BLASTP searches with an e-value of 1e^−5^, >40% identity value and >40% coverage. Domains of these genes were identified with hmmscan^115^ and further used to search against the proteome using hmmsearch^115^.

### Genes related to flavonoid biosynthesis

Genes related to flavonoid biosynthesis were retrieved from *A. thaliana* (KEGG: map00941) and homologs in the *R. nobile* genome were identified based on the constructed gene families. Homologs of several *bHLH* and *MYB* transcription factors that can activate multiple genes’ co-expression and play important roles in flavonoid biosynthesis were identified using iTAK software. Phylogenetic trees of *bHLH* and *MYB* proteins were constructed using iqtree.

### Antifreeze protein analysis

Antifreeze protein (*AFP*) improves an organisms’ ability to tolerate low temperatures, but has not been reported in the model plant *A. thaliana*. Thus, we identified homologs of *AFP*s from *Secale cereale* (Gene bank ID: AY590122, AY843521 and AY843522) and *Daucus carota* (Gene bank ID: A91926.1). These proteins were then used to search for homologs in the predicted proteome of *R. nobile* using BLASTP^84^ with an e-value of 1e^−5^, >40% identity value and >40% coverage. Moreover, HMM profiles of *LEA*s (LEA1, PF03760; LEA2, PF03168; LEA3, PF03242; LEA4, PF02987; LEA5, PF00477; LEA6, PF10714; Dehydrin, PF0025; SMP, PF04927) were downloaded from the Pfam database (http://pfam.sanger.ac.uk/) and HMMER^115^ was used to search the encoded protein sequences with default parameters and a filter threshold of 1e^−5^.

### Statistics and reproducibility

The functional enrichment analysis was performed using the ClusterProfile. The statistical significance of GO terms was evaluated using Fisher’s exact test in combination with FDR correction for multiple testing (*P* < 0.05). All experiments were carried out at least three times, independently, with similar results. All values are presented as means ± SD. Statistical significance was based on *t* tests. For RNA-seq and metabolite profiling, three biological repeats were used. For and chlorophyll and carotenoid concentrations, three biological replicates, each represented by three technical replicates from the same individual were used. And the software version and parameters applications in this study were listed in Supplementary Tables 17.

### Reporting summary

Further information on research design is available in the Nature Portfolio Reporting Summary linked to this article.

### Supplementary information

Supplemental material

Reporting Summary

## Data Availability

The genome assembly file and genome annotation files (contig level and chromosome level) are available at figshare (10.6084/m9.figshare.19662933). All genomic data (short-reads sequencing data, long-reads sequencing data and HiC sequencing data) and transcriptome data have been deposited at NCBI under the BioProject accession numbers of PRJNA830994 and PRJNA831329, respectively. The source data behind the graphs in Figs. 1d, 2a and 3d are available at Figshare (10.6084/m9.figshare.19662933) as Supplementary Data 2, 5 and 10, respectively. All other Supplementary Datas are also available at Figshare (10.6084/m9.figshare.19662933). All other data are available from the corresponding authors on reasonable request.
