# Supplementary material for: Multi-omics data provide insight into the adaptation of the glasshouse plant Rheum nobile to the alpine subnival zone
Source: Commun Biol. 2023 Sep 4;6:906. doi: 10.1038/s42003-023-05271-6 (PMC10477342; doi:10.1038/s42003-023-05271-6)
Supplement: Supplementary file 1 — Supplemental material [file 42003_2023_5271_MOESM1_ESM.pdf]

Supplementary Materials for

Adaptation and Evolution of the ‘Glasshouse’ Plant *Rheum nobile* in the Alpine Subnival Zone Based on Multi-omics Data

The file includes:

Supplementary Figures. 1 to 9

Supplementary Tables 1 to 17

Supplementary Figures

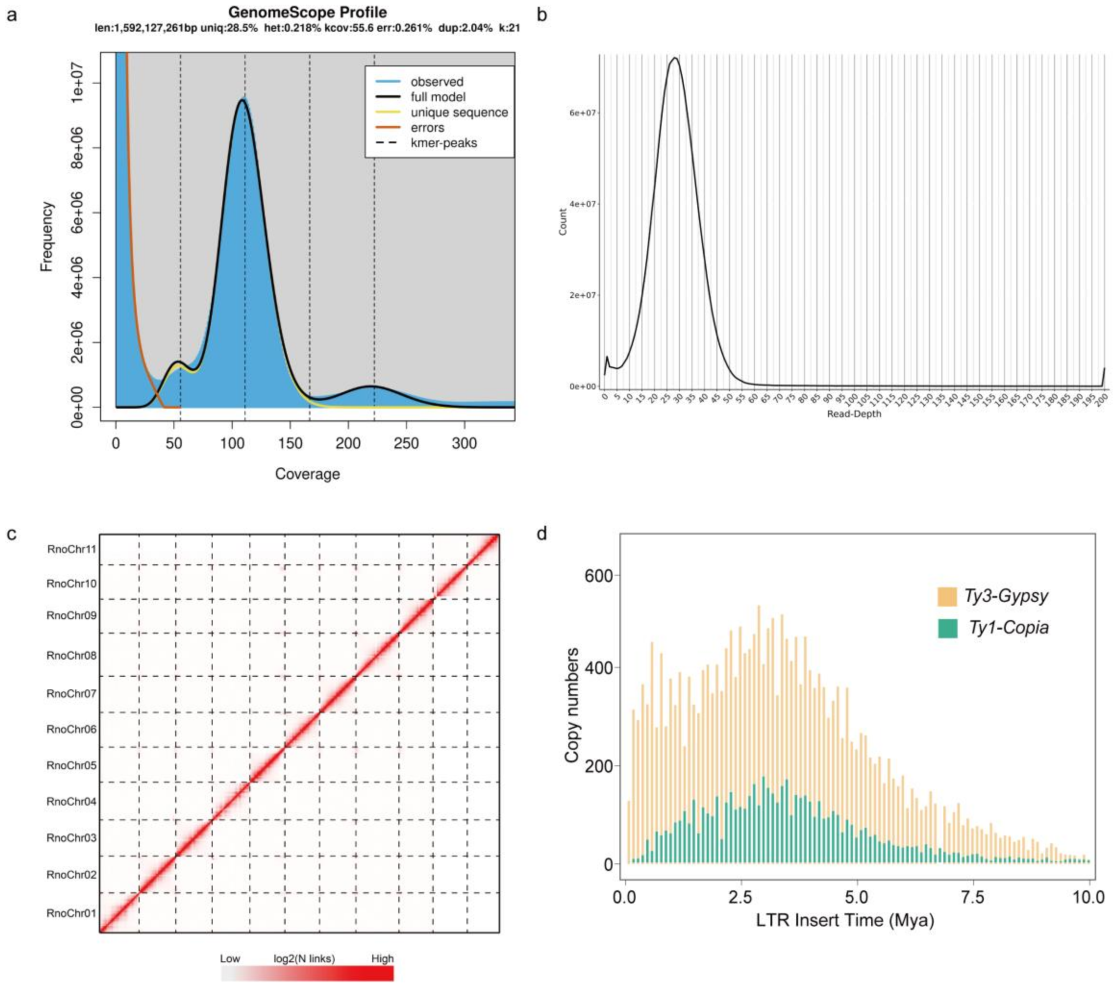

Supplementary Figure 1. Genome overview of noble rhubarb. a Genome size estimation based on GenomeScope with the *k*-mer size of 21; **b** Distribution of reads depth that calculated based on the result of the reads mapping to the contig-level assembly. **c** Genome-wide analysis of chromatin interactions in the chromosome-level genome assemblies of *R. nobile* based on Hi-C data (bin size = 500 kb); **d** Distribution of insertion times of *Copia* and *Gypsy* elements.

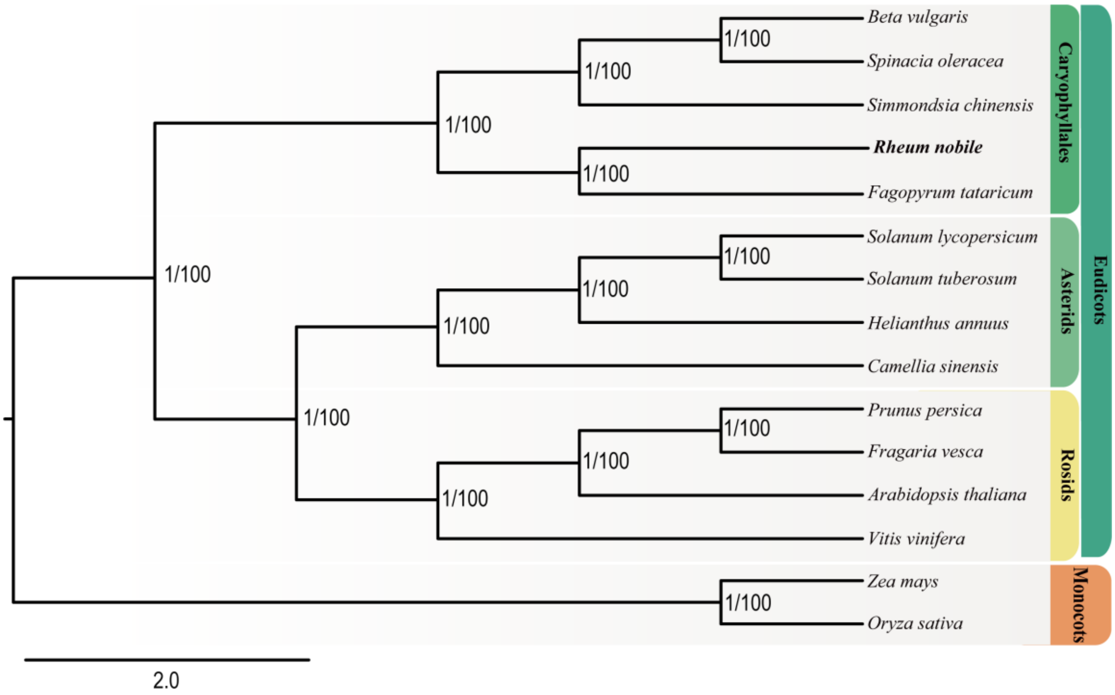

Supplementary Figure 2. Phylogenomic tree of noble rhubarb and 14 other species based on 1,098 single-copy gene families. Bootstrap support (left) values and posterior probabilities (right) are indicated with for each internal node. The coalescent-based and concatenation-based phylogeny trees showed the consisted topology with the high supporting values (posterior probability values equal to 1 and bootstrap values equal to 100).

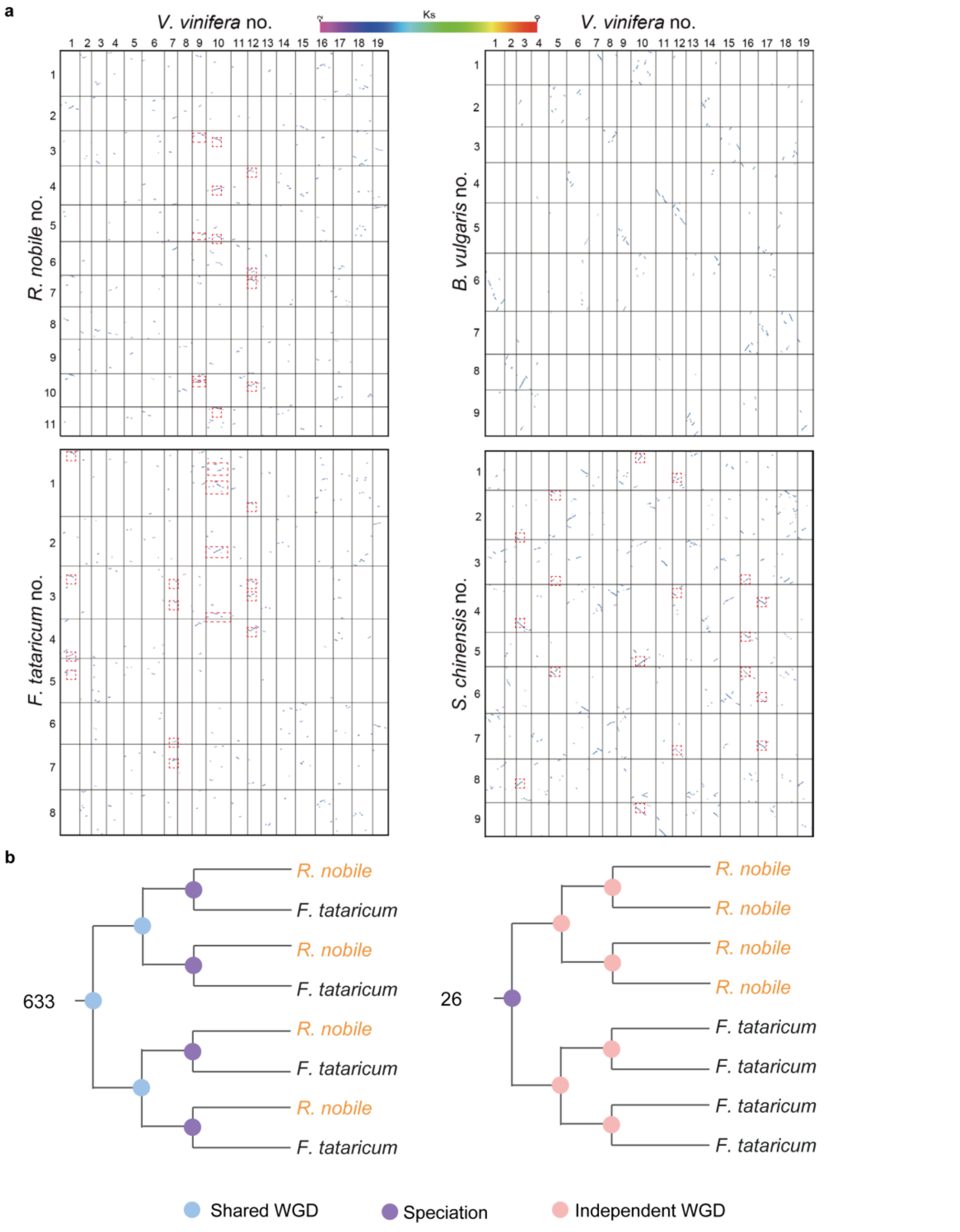

Supplementary Figure 3. Syntenic dotplots and collinear gene phylogeny analyses. a Phylogeny analyses of the collinear gene pairs between *R. nobile* and *F. tataricum*. The topologies corresponding to different scenario were showed, and the blue, pink and purple circle indicated “Shared WGD”, “Independent WGD” and “Speciation” in each node. The number at the left of each topology is the number of genes that supported corresponding topology; **b** The number of the genes that retained through two sharing WGDs of *R. nobile* and *F. tataricum*.

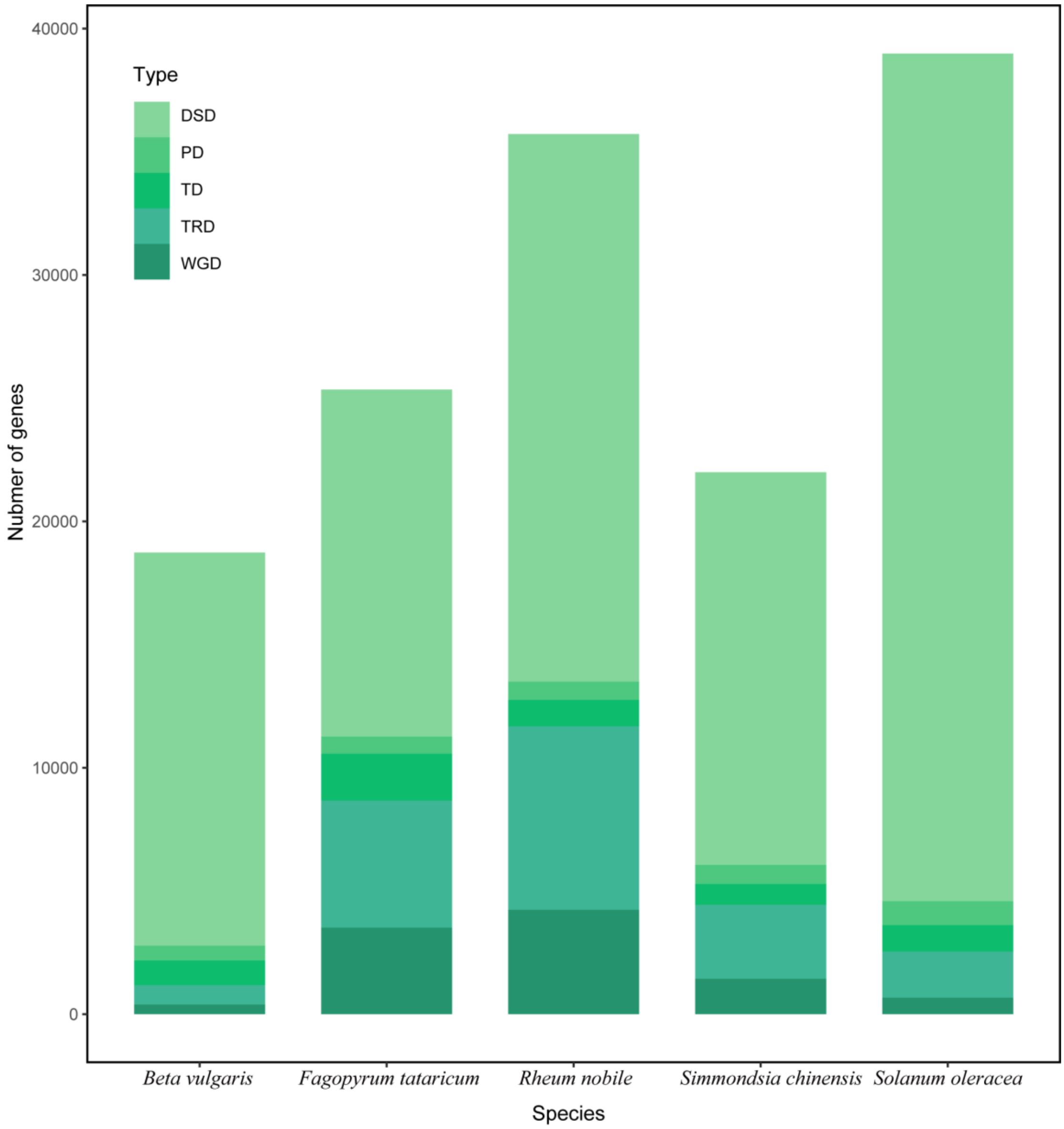

Supplementary Figure 4. Classification of gene duplicates origin in five Caryophyllales genome. The origins of gene duplicates were classified into five types: whole genome/segmental duplication (collinear genes in collinear blocks), tandem duplication (consecutive repeat), proximal duplication (two duplicated genes are distributed adjacent to each other on chromosomes, with no more than 10 genes spaced but not adjacent) and dispersed duplication (duplication type other than WGD/segmental, tandem and proximal).

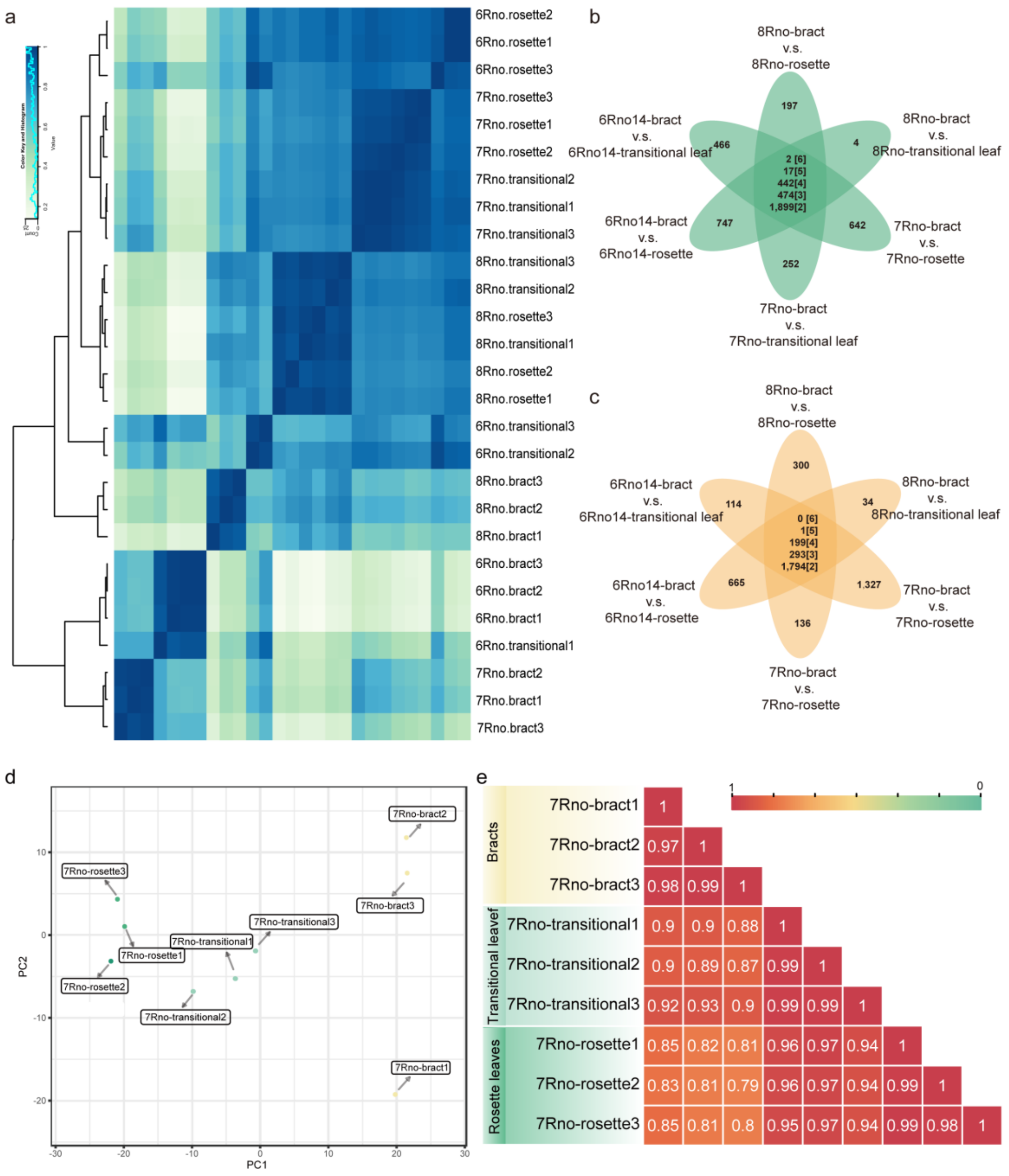

Supplementary Figure 5. Transcriptome and metabolome wide sample clustering analysis and the shared and specific different expression genes between three leaves. a The sample similarity matrix as a reflection of transcriptome wide gene expression; **b** and **c**, Venn diagram showing the shared and specific different downregulated and upregulated genes between three type leaves, respectively. Numbers in square bracket indicate number of groups with sharing different expression genes surviving duplicates; **d** PCA between the metabolomic samples of three type of leaves; **e** The sample similarity matrix represents the cumulative similarity of each sample as a reflection of metabolome wide type and content of large-scale metabolites.

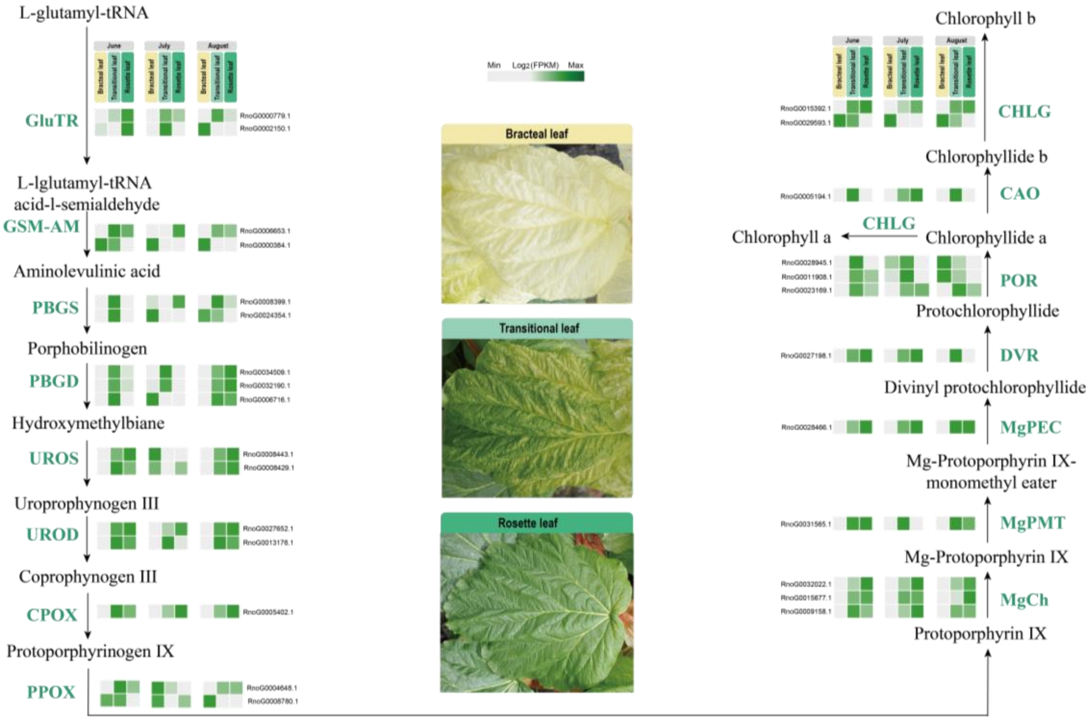

Supplementary Figure 6. The metabolic pathway and time-ordered gene regulation of Chlorophyll. Gene expression profile (in normalized FPKMs) in three types of leaf (bract, transitional leaf and rosette) at three time points in a growth season (June to August) were showed from left to right in each heatmap panel are presented in the heatmap alongside gene names. GluTR: Glutamyl-tRNA reductase; GSA-AM: L-Glutamate-1-semialdehyde2, 1-aminomutase; PBGS: Porphobilinogen synthase; PBGD: porphobilinogen deaminase; UROS: Uroporphyrinogen Ⅲ synthase; UROD: Uroporphyrinogen III decarboxylase; CPOX: Coproporphyrinogen III oxidase; PPOX: Protoporphyrinogen oxidase; MgCh: Magnesium chelatase H subunit; MgPMT: Magnesium proto IX methyltransferase; MgPEC: Mg-protoporphyrin IX monomethylester cyclase; POR: Protochlorophyllide oxidoreductase; CHLG: Chlorophyll synthase; CAO: Chlorophyllide a oxygenase.

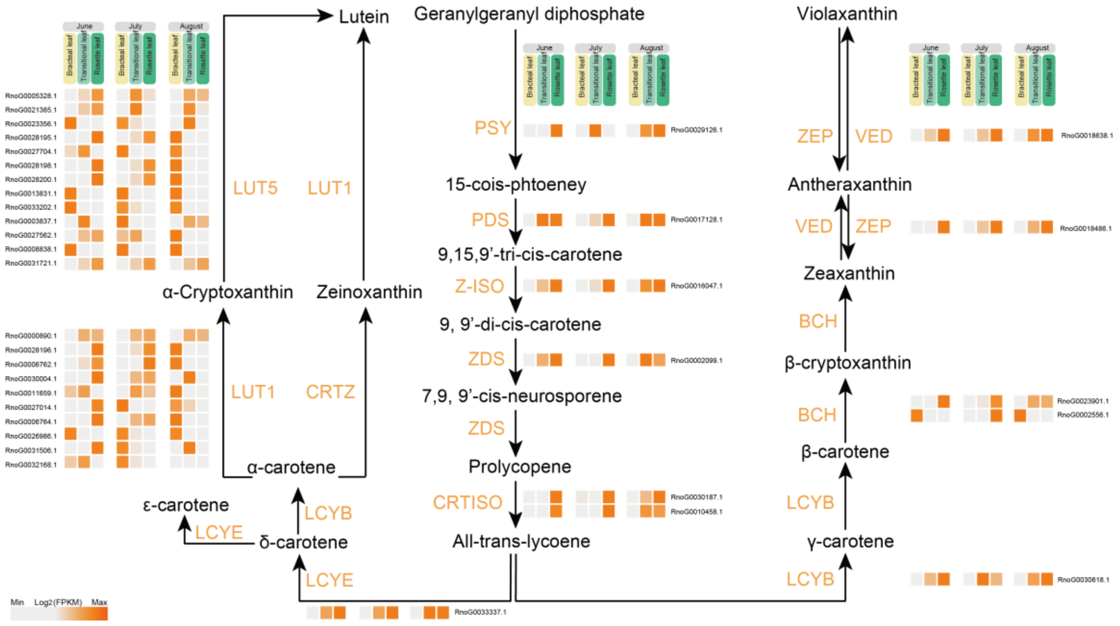

Supplementary Figure 7. The metabolic pathway and time-ordered gene regulation of carotenoids. Gene expression profile (in normalized FPKMs) in three types of leaf (bract, transitional leaf and rosette) at three time points in a growth season (here June, July and August). From left to right in each heatmap panel are presented in the heatmap alongside gene names. PSY: phytoene synthase; PDS: phytoene desaturase; Z-ISO: zeta-carotene isomerase; ZDS: zeta-carotene desaturase; CRTISO: carotene isomerase; LCYB: lycopene beta-cyclase; LCYE: lycopene δ-cyclase; CRTZ: beta-ring hydroxylase; BCH: beta-carotene hydroxylase; LUT1: carotene epsilon-monooxygenase; VDE: violaxanthin de-epoxidase (EC 1.10.99.3); ZEP: zeaxanthin epoxidase.

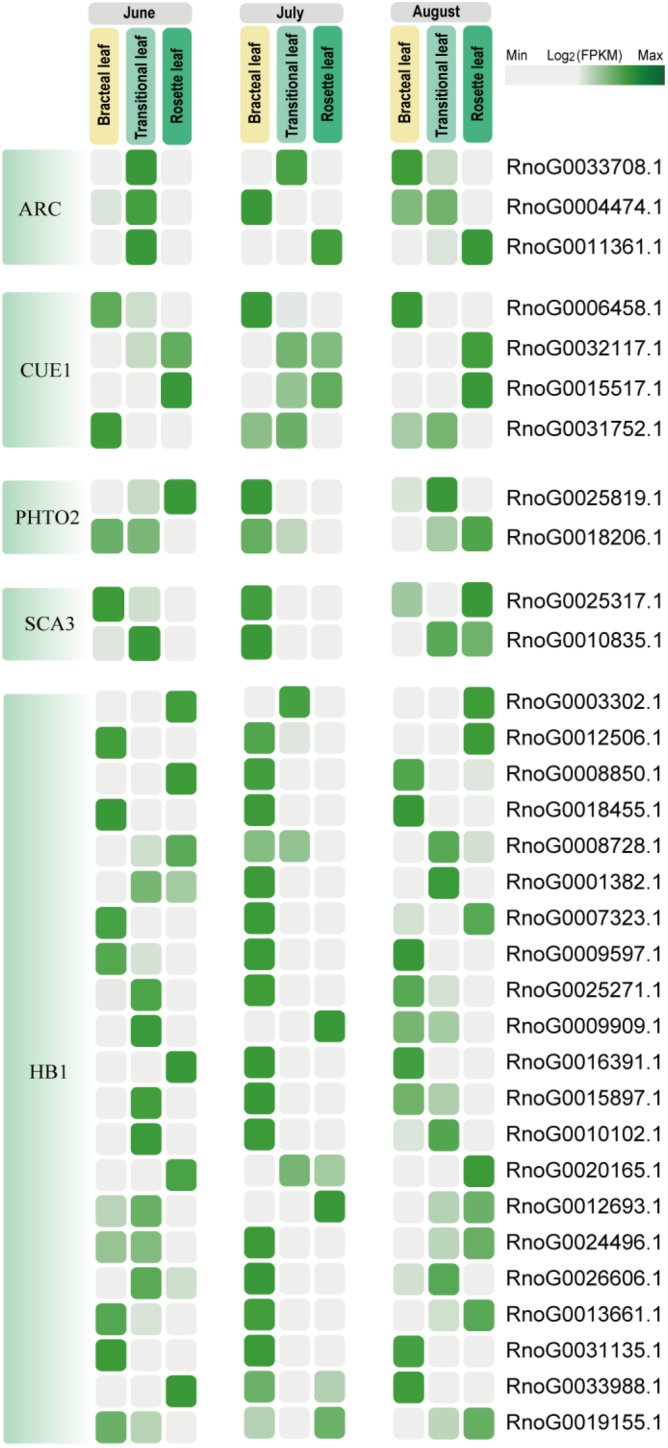

Supplementary Figure 8. Gene expression heatmap of genes that known to be involved in chloroplast and mesophyll development in leaves. The metabolic pathway and time-ordered gene regulation of carotenoids. Gene expression profile (in normalized FPKMs) in three types of leaf (bract, transitional leaf and rosette) at three time points in a growth season (here June-August (all sampled at 2 p.m.)) were showed from left to right in each heatmap panel are presented in the heatmap alongside gene names. ARC: accumulation and replication of chloroplasts; CUE1: CAB UNDEREXPRESSED 1; PHTO2: Phototropin 2; SCA3: SCABRA3; HB1: homeobox 1.

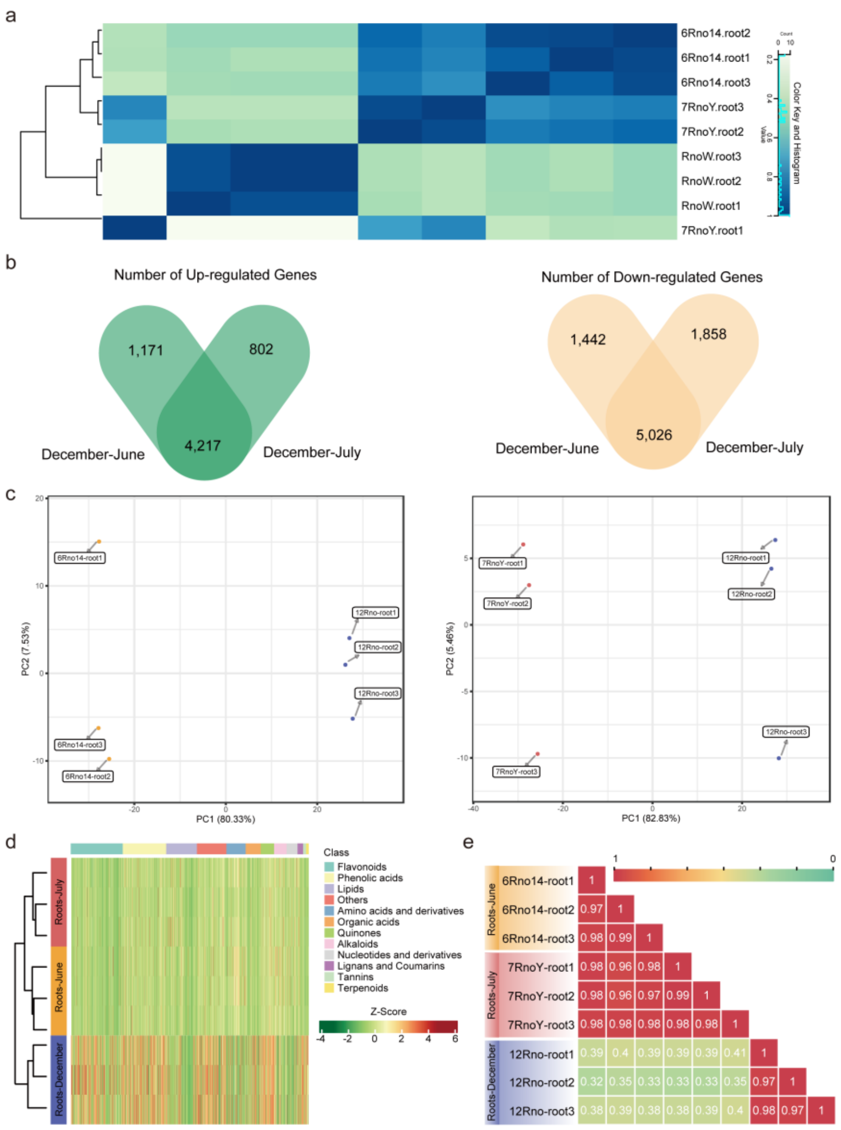

Supplementary Figure 9. Transcriptome and metabolome wide sample clustering analysis and the shared and specific different expression genes between the roots that sampled in three time points (June, July and December). a The sample similarity matrix represents the cumulative similarity of each sample at each time point as a reflection of transcriptome wide gene expression; **b** Venn diagram showing the shared and specific different upregulated (left) and downregulated (right). Numbers in square bracket indicate number of groups with sharing different expression genes surviving duplicates; **c** Subgroup principal component analysis between winter root and summer root (June and July); **d** The cluster dendrogram also groups samples based upon similar metabolites accumulation profiles; **e** The sample similarity matrix represents the cumulative similarity of each sample as a reflection of metabolome wide type and content of large-scale metabolites.

Supplementary Tables

Supplementary Table 1. Summary of the raw sequencing data.

Library Type	Base (Gb)	Depth (×)
Illumina	217.64	135
HiFi	46.63	29
Hi-C	189.62	119

* Depth (×) = base count / estimated genome size

Supplementary Table 2. Statistics for assembly and annotation of the draft gnome of noble rhubarb.

Assembly	*R. nobile*
**Genome size estimate (G)**	1.59
**Heterozygosity (%)**	0.22
**Genome assembly (G)**	1.57
Contig N50 (M)	6.72
Contig N90 (M)	1.34
Longest Contig (M)	36.72
Total Contig length (G)	1.57
**BUSCO (%)**	**97.5**
**Annotation**	
No. of predicted protein-coding genes	37,770
Average gene length (bp)	4314.35
Average CDS length (bp)	1076.45
Average exon per gene	4.97
Average length of exons (bp)	216.73
Average length of intron (bp)	816.23
Percentage of repeat sequence (%)	83.69
**BUSCO (%)**	**95.6**

Supplementary Table 3. Summary of chromosome-level assembles of noble rhubarb based on Hi-C data.

Chromosome	Number of anchored and oriented contigs	Length of anchored and oriented contigs (bp)	Number of anchored and oriented gene
Chr1	67	162,681,177	4,064
Chr2	32	143,605,298	3,086
Chr3	39	140,400,713	3,178
Chr4	34	140,007,204	3,521
Chr5	33	139,907,529	3,304
Chr6	31	137,450,686	3,041
Chr7	40	135,503,078	2,847
Chr8	35	133,562,642	2,918
Chr9	33	130,015,115	2,123
Chr10	55	126,994,042	2,989
Chr11	37	113,113,601	2,627

Supplementary Table 4. Numbers of Illumina short reads mapped to the assembled noble rhubarb genome.

species	Total pairs	Pair end mapped reads	Pair end mapped ratio	Single end mapped reads	Single end mapped ratio	Total mapped reads	Total mapped ratio
R.nobile	1,433,882,070	1,426,113,248	99.46	663,184	0.05	1,432,719,250	99.92

Supplementary Table 4. Quality assessment of the genome of *R. nobile* using QV scores and LAI index.

Chr	QV score	LAI
RnoChr01	50.36	28.26
RnoChr02	54.97	27.59
RnoChr03	53.94	27.87
RnoChr04	52.86	27.32
RnoChr05	50.71	27.05
RnoChr06	52.98	27.04
RnoChr07	50.69	27.15
RnoChr08	54.99	26.57
RnoChr09	51.79	27.65
RnoChr10	50.25	26.11
RnoChr11	51.43	28.93

Supplementary Table 5. Quality assessment of the assembled genome of noble rhubarb using BUSCOs.

Type	*R. nobile*	
	**Number**	**Percent (%)**
Complete BUSCOs (C)	1573	97.5
Complete and single-copy BUSCOs (S)	1499	92.9
Complete and duplicated BUSCOs (D)	74	4.6
Fragmented BUSCOs (F)	18	1.1
Missing BUSCOs (M)	23	1.4
Total BUSCO groups searched	1614	100

Supplementary Table 6. Summary statistics of the annotated transposable elements in the noble rhubarb genome.

Type	*R. nobile*		
	Length(bp)	% of repeat	% of genome
**SINE**	**2400939**	**0.18**	**0.15**
**LINE**	**52938666**	**4.02**	**3.37**
L1	51356320	3.90	3.27
L2	347986	0.03	0.02
LINE other	2064516	0.16	0.13
**LTR**	**943932202**	**71.75**	**60.05**
Copia	217894631	16.56	13.86
Gypsy	595782500	45.29	37.90
other	179051607	13.61	11.39
**DNA**	**99358122**	**7.55**	**6.32**
CMC-EnSpm	6984022	0.53	0.44
DNA other	92636138	7.04	5.89
Unclassified ARTEFACT	1406	0.00	0.00
Unclassified Other/Composite	631	0.00	0.00
Unclassified RC	2284	0.00	0.00
Unclassified RC/Helitron	17447988	1.33	1.11
Unclassified Retroposon	1702	0.00	0.00
Unclassified Retroposon/L1	179	0.00	0.00
Unclassified Unknown	209848501	15.95	13.35
**Satellite**	**7410735**	**0.56**	**0.47**
**Simple repeat**	**97463453**	**7.41**	**6.20**
**Small RNA**	**27900927**	**2.12**	**1.78**
**Total**	**1315561091**	**100.00**	**83.69**
**Low complexity**	**893929**	**0.07**	**0.06**

Supplementary Table 7. Prediction of protein-coding genes in the noble rhubarb genomes.

Gene set		Total Genes Predicted	Average Gene Length (bp)	Average CDS Length (bp)	Average Exons per Gene	Average Exon Length (bp)	Average Intron Length (bp)
De novo	augustus	32230	2579.39	932.53	4.75	196.51	439.43
	genescan	32230	23544.00	1137.61	6.22	182.93	4293.38
	glimmerhmm	32230	1367.82	630.82	2.98	211.38	371.41
Homolog	*Arabidopsis thaliana*	32230	3060.41	1198.36	5.66	211.79	399.73
	Beta vulgaris	43403	2759.49	1239.37	4.75	260.83	405.19
	Prunus persica	33811	3055.48	1201.88	5.59	215.03	403.89
	Spinacia oleracea	41385	2829.25	1253.72	4.90	255.95	404.15
	Vitis vinifera	34499	3041.14	1231.05	5.45	226.00	407.02
RNA-seq		28505	3813.01	988.06	4.61	214.49	451.75
**EVM**		**37770**	**4314.35**	**1076.45**	**4.97**	**216.73**	**816.23**

Supplementary Table 8. Quality assessment of the gene annotations of noble rhubarb using BUSCOs.

Type	*R. nobile*	
	**Number**	**Percent (%)**
Complete BUSCOs (C)	1543	95.6
Complete and single-copy BUSCOs (S)	1476	91.4
Complete and duplicated BUSCOs (D)	67	4.2
Fragmented BUSCOs (F)	31	1.9
Missing BUSCOs (M)	40	2.5
Total BUSCO groups searched	1614	100

Supplementary Table 9. Functional annotation of predicted protein-coding genes in the noble rhubarb genome.

Database	Number of genes annotated	Percentage (%)
GO	25768	68.18
KEGG	19458	51.48
SWISS-PROT	26912	71.20
TrEMBL	33421	88.42
NR	33539	88.74
Total	34270	90.68

Supplementary Table 10. Detection of transcription factors in the noble rhubarb genomes.

Transcription factor	*R. nobile*	Transcription factor	*R. nobile*	Transcription factor	*R. nobile*
LFY	2	RB	1	SET	50
HSF	0	HB-KNOX	9	B3-ARF	37
CAMTA	5	DDT	4	TRAF	29
MADS-MIKC	3	MYB	136	SWI/SNF-SWI3	4
MADS-M-type	12	SNF2	39	Others	125
VOZ	3	Tify	13	mTERF	48
FAR1	1	GNAT	46	TAZ	5
LIM	6	PHD	35	B3	74
SAP	2	NAC	98	BES1	12
C2C2-LSD	4	STAT	1	BBR-BPC	5
DBB	4	EIL	9	OFP	21
TCP	16	NF-YA	12	LUG	4
zf-HD	18	SBP	20	HMG	15
Coactivator	4	SWI/SNF-BAF60b	19	HB-other	15
Pseudo	5	WRKY	74	bZIP	97
DBP	1	bHLH	154	C3H	72
C2C2-CO-like	11	GeBP	25	ULT	1
NF-YB	13	AP2/ERF-ERF	153	SRS	10
HB-BELL	11	AP2/ERF-AP2	20	ARID	18
GRF	9	MED7	1	Alfin-like	5
HB-PHD	2	C2C2-Dof	36	AP2/ERF-RAV	3
Whirly	2	HB-HD-ZIP	44	E2F-DP	7
GARP-ARR-B	11	SOH1	1	MYB-related	79
C2C2-YABBY	8	GRAS	37	TUB	15
HB-WOX	15	PLATZ	16	Rcd1-like	1
CSD	1	Trihelix	46	NOZZLE	0
BSD	1	GARP-G2-like	60	AUX/IAA	41
RWP-RK	14	C2C2-GATA	34	MED6	1
IWS1	11	MBF1	3	S1Fa-like	1
NF-YC	7	NF-X1	4	CPP	7
LOB	48	Jumonji	21	**Total**	**2341**
HRT	1	C2H2	117		

Supplementary Table 11. Prediction of noncoding RNAs in the noble rhubarb genomes.

Type	Total number	Average length (bp)	Total length (bp)
antisense	8	191.50	1,532
miRNA	101	128.60	12,989
rRNA	6824	145.31	991,566
ribozyme	5	141.20	706
sRNA	2	306.00	612
snRNA	1,231	116.74	143,704
tRNA	2,776	72.02	199,927

Supplementary Table 12. The samples information related to investigate the genetic mechanism of the structure difference between bracts and rosette leaves. The "*" indicated the sample used both in transcriptomic and metabolomic analyses.

Tissue	Sample ID	Sampling Time	Number of Reads	Number of Mapped Reads	Mapping Rate
Bract	6Rno-bract1	June 6, 2021	86,030,252	77,517,585	90.11%
	6Rno-bract2		84,578,270	75,858,481	89.69%
	6Rno-bract3		85,529,574	76,861,341	89.87%
	*7Rno-bract1	July 6, 2021	58,960,688	50,650,398	85.91%
	*7Rno-bract2		57,515,903	50,298,817	87.45%
	*7Rno-bract3		118,447,836	104,725,272	88.41%
	8Rno-bract1	August 19, 2021	45,837,317	44,872,518	97.90%
	8Rno-bract2		45,449,275	44,428,288	97.75%
	8Rno-bract3		45,121,241	44,046,015	97.62%
Transitional leaf	6Rno-transitional1	June 6, 2021	80,429,608	72,336,974	89.94%
	6Rno-transitional2		87,181,504	78,421,037	89.95%
	6Rno-transitional3		84,835,163	77,033,854	90.80%
	*7Rno-transitional1	July 6, 2021	96,937,241	86,901,963	89.65%
	*7Rno-transitional2		83,694,654	75,350,693	90.03%
	*7Rno-transitional3		101,527,447	91,520,376	90.14%
	8Rno-transitional1	August 19, 2021	44,937,577	43,733,976	97.32%
	8Rno-transitional2		44,339,055	43,325,513	97.71%
	8Rno-transitional3		43,986,335	42,949,185	97.64%
Rosette leaf	6Rno-rosette1	June 6, 2021	48,145,587	43,752,674	90.88%
	6Rno-rosette2		99,005,260	89,953,515	90.86%
	6Rno-rosette3		97,588,917	88,453,076	90.64%
	*7Rno-rosette1	July 6, 2021	91,458,064	81,934,985	89.59%
	*7Rno-rosette2		97,029,258	86,714,895	89.37%
	*7Rno-rosette3		78,261,711	70,323,380	89.86%
	8Rno-rosette1	August 19,2021	44,660,932	43,612,976	97.65%
	8Rno-rosette2		44,332,151	43,314,201	97.70%
	8Rno-rosette3		44,182,031	43,082,404	97.51%

Supplementary Table 13. The annotation information of the mature miRNA that similarity with the miRNAs involved in regulating flavonoid biosynthesis in *Arabidopsis*.

Chr	Start	Len	Sequence	miRBase	SVM probability
RnoChr07	403,921	21	UCGGACCAGGCUUCAUUCCCC	miR166	0.97
RnoChr02	103,181,122	21	UCGGACCAGGCUUCAUUCCCC	miR166	0.94
RnoChr03	136,022,870	21	UAUUGGUGAGGUUCAAUCCGA	miR171	0.92
RnoChr01	123,876,771	21	AGAUUGAGCCGCGCCAAUAUC	miR171	0.94
RnoChr03	136071316	21	UUCCACAGCUUUCUUGAACUU	miR396	0.87

Supplementary Table 14. The samples information related to investigate adaptive basis of overwintering in noble rhubarb. The "*" indicated the sample used both in transcriptomic and metabolomic analyses.

**Sample ID**	**Sample Time**	**Number of Reads**	**Number of Mapped Reads**	**Mapping Rate**
*6Rno14-root1	June 6, 2021	67,502,277	59,188,190	87.68%
*6Rno14-root2		62,573,535	55,041,086	87.96%
*6Rno14-root3		31,213,123	27,414,468	87.83%
*7RnoY-root1	July 6, 2021	74,880,365	66,875,410	89.31%
*7RnoY-root2		79,019,860	69,747,036	88.27%
*7RnoY-root3		9,199,597	8,046,357	87.46%
*12Rno-root1	December 5, 2021	84,081,760	79,692,520	94.78%
*12Rno-root2		91,821,536	84,899,742	92.46%
*12Rno-root3		88,218,799	83,057,442	94.15%

Supplementary Table 15. Comparison of genome assembly metrics for different versions of *R. nobile* genome.

**Metrics**	**This study**	**Previous version**
Genome size estimate (G)	1.59	1.48
Genome assembly (G)	1.57	1.36
Contig N50 (M)	6.72	9.8
Longest Contig (M)	36.72	23.56
BUSCO (%)	97.5	94.5
QV Score	51.96	46.3
LAI	25.08	9.9
Mapping rate (%)	99.92	99.37
**Chromosome Number**	11	-
No. of predicted protein-coding genes	37,770	58,950

Supplementary Table 16. Reference information of the genomes used in this study.

Species	Version	Source
Beta vulgaris	RefBeet-1.2.2	NCBI
Solanum oleracea	v1.0	NCBI
Fagopyrum tataricum	Pinku1	http://mbkbase.org/Pinku1
Simmondsia chinensis	v1.0	ftp://download.big.ac.cn
Fragaria vesca	v4.0.a2	ftp://ftp.bioinfo.wsu.edu
Prunus persica	v2	NCBI
Arabidopsis thaliana	TAIR10.1	NCBI
Vitis vinifera	12X	NCBI
Camellia sinensis	HZAU_G240_1.0	NCBI
Solanum lycopersicum	v3.0	NCBI
Solanum tuberosum	v3.0	NCBI
Helianthus annuus	HanXRQr2.0	NCBI
Oryza sativa	IRGSP-1.0	NCBI
Zea mays	NAM-5.0	NCBI
Rheum nobile	v1.0	This study

Supplementary Table 17. The software version and parameters applications in this study.

Software and Algorithms	Parameters
fastp (v.0.20.0)	default
Jellyfish (v.2.2.10)	jellyfish count /dev/fd/0 -C -o Cma_21mer -m 21 -t 48 -s 50G; jellyfish histo -h 5000000 -o Cma_21mer.histo Cma_21mer
GenomeScope (v1.0.0)	genomescope.R Cma_out.histo 21 150 Cma_21mer
hifiasm (v. 0.15.3-r339)	--h1 HiC.R1.fastq.gz --h2 HiC.R2.fastq.gz Rno.hifi.fq.gz 2>&1 | tee Rno.HiC.asm.log
Mequry	meryl k=21 count output Rno.1.meryl Rno.1.ngs.merge.fq.gz<br>meryl k=21 count output Rno.2.meryl Rno.2.ngs.merge.fq.gz<br>meryl union-sum output Rno.meryl Rno.*.meryl<br>merqury.sh Rno.meryl Rno.genome.fa Rno
LTR_retriver	default
Purge_Haplotigs	default
Purge_dup	default
BUSCO (v.3)	embryophyta_odb10 -m genome -c 20 -sp arabidopsis
HiCUP	default
3D-DNA	default
RepeatMasker	-nolow -norna -no_is -gff -species Mesangiospermae
RepeatProteinMasker	-noLowSimple -pvalue 1e-04
RepeatModeler	default
LTR_Finder (v1.06)	default
bedtools (v.2.29.2)	bedtools merge -i All.repeat.bed > All.repeat.merge.bed; bedtools maskfasta -fi curated.fasta -bed All.repeat.merge.bed -fo curated.fasta.mask
Augustus (v.3.2.3)	--species=arabidopsis
GenScan	Arabidopsis.smat
GlimmerHMM (v.3.0.4)	-d arabidopsis
GoMoMa (v.1.6)	AnnotationFinalizer.r=NO tblastn=false
Trinity (v.2.1.1)	--seqType fq --max_memory 120G --CPU 30 --full_cleanup --trimmomatic --normalize_reads
PASA (v2.4.1)	Launch_PASA_pipeline.pl -c pasa.config -C -R -g genome.fasta -t Trinity.fasta --ALIGNERS blat,gmap --CPU 30
EVidenceModeler (v.1.1.1)	evidence_modeler.pl -G genome.fasta -g ab_initio.gff -w evm.weights.txt -e rna_seq.gff -p homolog.gff --exec_dir evm_out > evm.out
Infernal (v.1.1.2)	cmscan -cut_ga --rfam --nohmmonly --tblout result/Rno.tblout --fmt 2 --clanin Rfam.clanin Rfam.cm genome.fa > genome.cmscan

Continued Supplementary Table 17. The software version and parameters applications in this study.

Software and Algorithms	Parameters
miRPara (v6.0)	default
psRoBot (v1.2)	default
BLASTP (v.2.7.1+)	E-value < 1×10^{-5} -outfmt 7 -num_threads 20
InterProScan (v.5.28)	interproscan.sh -appl TIGRFAM,ProDom,Hamap,SMART,ProSiteProfiles,ProSitePatterns,SUPERFAMILY,PRINTS,Gene3D,PIRSF,Pfam,Coils -f tsv -iprlookup -goterms -pa -tp -td ./temp
Hmmer (v3.1b2)	default
OrthoMCL (v.2.0.9-4)	default
iqtree (v.2.0.3)	-s concatenation.fa -st DNA -pre concatenation.fa -nt 5 -bb 1000 -m MFP -quiet -redo
PAML (v.4.8)	baseml baseml.ctl; mcmctree mcmctree.ctl > mcmctree.log
CAFÉ (v.2.2)	cafe2.2 cafetutorial_run1.sh
MCScanX	python -m jcvi.formats.gff bed --type=mRNA --key=ID genome.gff -o Sp.bed; python -m jcvi.compara.catalog ortholog Sp1 Sp2; python -m jcvi.compara.synteny screen --minspan=30 --simple lifted.anchors anchors.new; python -m jcvi.graphics.karyotype seqids layout
WGDI	default
HISAT2 (v.2.2.1)	-x Rno.genome -p 20 -X 500 --fr --min-intronlen 20 --max-intronlen 500000 --dta -1 Rno.leaf.trim.1.fq.gz -2 Rno.leaf.trim.2.fq.gz 2> 8Rno-jibuye3.log |samtools view -bS - | samtools sort - -o Rno.leaf.sort.bam
StringTie (v.2.1.2)	-e -G Rno.genomic.gff -p 20 -B -o Rno.leaf.sort.bam.gtf Rno.leaf.sort.bam
DEseq2 (v.1.22.2)	default
ClusterProfiler	default
