# Supplementary material for: Multi-omics data provide insight into the adaptation of the glasshouse plant Rheum nobile to the alpine subnival zone
Source: Commun Biol. 2023 Sep 4;6:906. doi: 10.1038/s42003-023-05271-6 (PMC10477342; doi:10.1038/s42003-023-05271-6)
Supplement: Supplementary file 2 — Reporting Summary [file 42003_2023_5271_MOESM2_ESM.pdf]

nature portfolio

Corresponding author(s): Yongzhi Yang

Last updated by author(s): Aug 14, 2023

Reporting Summary

Nature Portfolio wishes to improve the reproducibility of the work that we publish. This form provides structure for consistency and transparency in reporting. For further information on Nature Portfolio policies, see our Editorial Policies and the Editorial Policy Checklist.

Statistics

For all statistical analyses, confirm that the following items are present in the figure legend, table legend, main text, or Methods section.

n/a	Confirmed	
☐	☒	The exact sample size (*n*) for each experimental group/condition, given as a discrete number and unit of measurement
☒	☐	A statement on whether measurements were taken from distinct samples or whether the same sample was measured repeatedly
☐	☒	The statistical test(s) used AND whether they are one- or two-sided<br>*Only common tests should be described solely by name; describe more complex techniques in the Methods section.*
☐	☒	A description of all covariates tested
☐	☒	A description of any assumptions or corrections, such as tests of normality and adjustment for multiple comparisons
☐	☒	A full description of the statistical parameters including central tendency (e.g. means) or other basic estimates (e.g. regression coefficient) AND variation (e.g. standard deviation) or associated estimates of uncertainty (e.g. confidence intervals)
☐	☒	For null hypothesis testing, the test statistic (e.g. *F*, *t*, *r*) with confidence intervals, effect sizes, degrees of freedom and *P* value noted<br>*Give P values as exact values whenever suitable.*
☐	☒	For Bayesian analysis, information on the choice of priors and Markov chain Monte Carlo settings
☐	☒	For hierarchical and complex designs, identification of the appropriate level for tests and full reporting of outcomes
☐	☒	Estimates of effect sizes (e.g. Cohen's *d*, Pearson's *r*), indicating how they were calculated

Our web collection on statistics for biologists contains articles on many of the points above.

Software and code

Policy information about availability of computer code

Data collection	No software was used to collect the data
Data analysis	Genome size estimation: Jellyfish v.2.2.10 and GenomeScope v.1.0.0<br>Genome assembly: fastp v.0.20.0, Hifiasm 0.15.3-r339, BWA-MEM 0.7.10-r789 and 3D-DNA v.180922<br>Genome quality estimation: RepeatMasker, BUSCO, mequry<br>Genome annotation: RepeatMasker v.4.1.0, RepeatProteinMasker v.4.1.0, LTR_Finder v.1.06, Augustus v.3.2.3, GenScan, GlimmerHMM v.3.0.4, EVidenceModeler v.1.1.1, PASA, BLASTP v.2.7.1+, InterProScan v.5.28, iTAK,INFERNAL v1.1.2<br>Phylogenetic analysis: BLASTP v.2.7.1+, OrthoMCL v. 2.0.9-4, IQ-TREE v. 2.0.3-h176a8bc_0, ASTRAL, MCMCtree v.4.8, PAML package v.4.8 and CAFÉ v.4.2.<br>WGD Detection: WGDI v.0.5.3

For manuscripts utilizing custom algorithms or software that are central to the research but not yet described in published literature, software must be made available to editors and reviewers. We strongly encourage code deposition in a community repository (e.g. GitHub). See the Nature Portfolio guidelines for submitting code & software for further information.

Data

Policy information about availability of data

All manuscripts must include a data availability statement. This statement should provide the following information, where applicable:

- Accession codes, unique identifiers, or web links for publicly available datasets
- A description of any restrictions on data availability
- For clinical datasets or third party data, please ensure that the statement adheres to our policy

The genome assembly file and genome annotation files (contig level and chromosome level) are available at figshare (10.6084/m9.figshare.19662933). All genomic data (short-reads sequencing data, long-reads sequencing data and HiC sequencing data) and transcriptome data have been deposited at NCBI under the BioProject accession numbers of PRJNA830994 and PRJNA831329 , respectively. The reviewer link of these three BioProject were listed below: https://dataview.ncbi.nlm.nih.gov/object/PRJNA831329?reviewer=k983uv8is2kh16i18d636cr4p0 and https://dataview.ncbi.nlm.nih.gov/object/PRJNA745478?reviewer=vb2u9ue9sd5c16jk0ov6tpir1t.

Human research participants

Policy information about studies involving human research participants and Sex and Gender in Research.

Reporting on sex and gender	N/A
Population characteristics	N/A
Recruitment	N/A
Ethics oversight	N/A

Note that full information on the approval of the study protocol must also be provided in the manuscript.

Field-specific reporting

Please select the one below that is the best fit for your research. If you are not sure, read the appropriate sections before making your selection.

☒ Life sciences ☐ Behavioural & social sciences ☐ Ecological, evolutionary & environmental sciences

For a reference copy of the document with all sections, see nature.com/documents/nr-reporting-summary-flat.pdf

Life sciences study design

All studies must disclose on these points even when the disclosure is negative.

Sample size	One mature Rheum nobile individual was collected in the field and used to the genome sequencing. The different tissues (Bracteal leaf, transitional leaf, rosette leaf, root and flower) from other individuals in different stage (June, July, August and December) were collected and used for transcriptome and metabolomic sequencing.
Data exclusions	For the long Hifi reads, we don't perform further filtering. For the Illumina short reads (genomic or transcriptome), the low quality reads were filtered by fastp with default parameters.
Replication	During transcriptomes and metabolomic , each tissue had three replicates.
Randomization	No randomization in our analysis. Within the genomic, transcriptome and metabolomics analyses, randomization is not needed. For the phylogeny analysis, all orthologous genes were used, so no randomization needed.
Blinding	We collected the samples in the wild and sequenced and assembled the genome with no control group is referred here. Blinding is not applicable in this study.

Reporting for specific materials, systems and methods

We require information from authors about some types of materials, experimental systems and methods used in many studies. Here, indicate whether each material, system or method listed is relevant to your study. If you are not sure if a list item applies to your research, read the appropriate section before selecting a response.

Materials & experimental systems

n/a	Involved in the study
☒	☐ Antibodies
☒	☐ Eukaryotic cell lines
☒	☐ Palaeontology and archaeology
☒	☐ Animals and other organisms
☒	☐ Clinical data
☒	☐ Dual use research of concern

Methods

n/a	Involved in the study
☒	☐ ChIP-seq
☒	☐ Flow cytometry
☒	☐ MRI-based neuroimaging
